# Design and validation of an open-source modular Microplate Photoirradiation System for high-throughput photobiology experiments

**DOI:** 10.1371/journal.pone.0203597

**Published:** 2018-10-05

**Authors:** Suzanna Katz, Peter Backeris, Christopher Merck, Maria Suprun, Sunita D’Souza, David F. Bishop, Robert J. Desnick, Kateri Moore, Iban Ubarretxena-Belandia, Ihor R. Lemischka

**Affiliations:** 1 Department of Cell, Developmental and Regenerative Biology, Black Family Stem Cell Institute, Icahn School of Medicine at Mount Sinai, New York, NY, 10029 United States of America; 2 Department of Neurosurgery, Sinai Biodesign, Icahn School of Medicine at Mount Sinai, New York, New York, 10029 United States of America; 3 Merck Engineering LLC, Hamburg, NJ, 07495 United States of America; 4 Department of Pediatrics, Allergy and Immunology, Icahn School of Medicine at Mount Sinai, New York, NY, USA, Icahn School of Medicine at Mount Sinai, New York, NY, 10029 United States of America; 5 Department of Genetics and Genomic Sciences, Icahn School of Medicine at Mount Sinai, New York, NY, USA, Icahn School of Medicine at Mount Sinai, New York, NY, 10029 United States of America; 6 Department of Pharmacological Sciences, Icahn School of Medicine at Mount Sinai, New York, NY, United States of America; 7 Biofisika Institute (CSIC, UPV/EHU), University of the Basque Country, Leioa, Spain; Massachusetts General Hospital, UNITED STATES

## Abstract

Research in photobiology is currently limited by a lack of devices capable of delivering precise and tunable irradiation to cells in a high-throughput format. This limits researchers to using expensive commercially available or custom-built light sources which make it difficult to replicate, standardize, optimize, and scale experiments. Here we present an open-source Microplate Photoirradiation System (MPS) developed to enable high-throughput light experiments in standard 96 and 24-well microplates for a variety of applications in photobiology research. This open-source system features 96 independently controlled LEDs (4 LEDs per well in 24-well), Wi-Fi connected control and programmable graphical user interface (GUI) for control and programming, automated calibration GUI, and modular control and LED boards for maximum flexibility. A web-based GUI generates light program files containing irradiation parameters for groups of LEDs. These parameters are then uploaded wirelessly, stored and used on the MPS to run photoirradiation experiments inside any incubator. A rapid and semi-quantitative porphyrin metabolism assay was also developed to validate the system in wild-type fibroblasts. Protoporphyrin IX (PpIX) fluorescence accumulation was induced by incubation with 5-aminolevulinic acid (ALA), a photosensitization method leveraged clinically to destroy malignant cell types in a process termed *photodynamic therapy* (PDT), and cells were irradiated with 405nm light with varying irradiance, duration and pulsation parameters. Immediately after light treatment with the MPS, subsequent photobleaching was measured in live, adherent cells in both 96-well and a 24-well microplates using a microplate reader. Results demonstrate the utility and reliability of the Microplate Photoirradiation System to irradiate cells with precise irradiance and timing parameters in order to measure PpIx photobleaching kinetics in live adherent cells and perform comparable experiments with both 24 and 96 well microplate formats. The high-throughput capability of the MPS enabled measurement of enough irradiance conditions in a single microplate to fit PpIX fluorescence to a bioexponential decay model of photobleaching, as well as reveal a dependency of photobleaching on duty-cycle—but not frequency—in a pulsed irradiance regimen.

## Introduction

Advances in photobiology, especially in the fields of optogenetics and photomedicine, have widened interest in studying the effects of isolated spectra of light on cells in a controlled environment. In vitro photobiology experiments have been challenging to perform in a standardized and high-throughput manner, in part due to a lack of appropriate illumination technology compatible with multiwell experiments. Commercially available lamps, laser, LED, and microscope systems used as irradiation sources in these experiments are typically not designed for in vitro cell culture format. In addition, they are often expensive, cumbersome to use, have fixed spectral components, and lack the ability to irradiate individual cell culture wells in standard microplates with independently controllable timing and irradiance. Several groups have attempted to address some of these issues by developing custom LED arrays for irradiating cells in multiwell plates [1–3], while more recent attempts have resulted in systems capable of managing individual or grouped wells through a computer interface [4–7]. However, none of these systems contain all the key features listed below necessary for a truly high-throughput, reproducible and highly-configurable photoirradiation system suitable for a wide range of photobiology experiments in standard microplates:

- Able to work with both 24-well and 96-well plates to obtain the benefits of using either for a particular type of experiment/assay
- Able to provide individual wells in either format with homogeneous, calibrated LED irradiance and precise timing control, including pulsed irradiance patterns
- Fits readily inside a standard cell culture incubator
- Is customizable, modular, low-cost, and easy to assemble/repair
- Is reliable and easy to use
- Open-source design to facilitate easier reproducibility, modification and improvement by other groups

One area of photobiology research that could benefit from such a system is the study of photosensitization, a process in which photosensitive molecules (termed *photosensitizers*) are able to create a change within a biological system due to their absorption of a photon [8]. Porphyrins are tetrapyrrole macrocycles that are intermediate products in the highly conserved heme biosynthetic pathway and intrinsic photosensitizers to biological systems [9]. All tissue types are able to synthesize porphyrins and heme, but steady-state production of heme and the levels of the various porphyrin intermediates is cell-type specific. In the cells of the fetal liver and bone marrow, there is a large demand for heme production, and its intermediate Uroporphyrinogen III (the first in a series of tetrapyrrole intermediates) is used as a substrate in the eventual formation of heme. In other cell types, the enzymatic action of ALA synthase is inhibited by the presence of heme, thus regulating ALA production through negative feedback [9, 10].

The introduction of exogenous ALA that is not created by ALA synthase allows the downstream steps in the pathway to proceed uninhibited and recapitulates heme metabolism with its characteristic production of photosensitive intermediates, mainly the temporary accumulation of Protoporphyrin IX (PpIX) in the mitochondria. The accumulated PpIX can undergo a chemical reaction with molecular oxygen when excited with certain wavelengths of light to produce the cytotoxic singlet oxygen. This reaction has been exploited clinically in a process termed *Photodynamic Therapy* (PDT) to treat a number of tumors and skin malignancies. The precursor ALA can be applied topically or injected for the purpose of sensitizing and destroying aberrant cell types. Furthermore, it has been shown to have selective photoinactivation—and to preferentially accumulate—in metabolically active and abnormally proliferative cells (carcinomas, leukemia, gliomas, and various other cancers) [11–16], in malignant and non-malignant skin diseases (like scleredoma, actinic keratosis, acne vulgaris, and photo-damage) [17–25], and in pathogens like blood-borne and enveloped viruses or extracellular bacteria [26–34]. The ALA-treated area is subjected to light therapy after an incubation period and the photosensitive PpIX produces a local cytotoxic response in the targeted cells.

Due to the conserved nature of the pathway, ALA-induced PpIX can be used as a model system to study the mechanisms of photosensitivity in cells that do not otherwise synthesize heme but are more accessible and amenable to in vitro manipulation. Since heme and porphyrin metabolism dysregulation has been reported for a number of conditions like Alzheimer’s Disease, toxic metal poisoning like mercury and lead, arsenic carcinogenicity, the Porphyrias, Hepatitis C infection, chronic renal failure, gliomas, and many other cancer types [35–37], creating an in vitro platform where heme and porphyrin metabolism can be assayed rapidly and in a high-throughput and low-cost manner is in itself a valuable tool. Thus, we additionally describe a microplate assay that utilizes the ALA-induced PpIX model to showcase the capabilities of the MPS in live, adherent cells. This assay provides biological insights into ALA-induced photosensitivity in an in vitro model of PDT.

## Design and fabrication of the Microplate Photoirradiation System

A modular, programmable Microplate Photoirradiation System (MPS) was developed to enable precise, highly-configurable and high-throughput studies of the effects of LED light on cells in vitro using standard 96 or 24-well microplates. The system comprises three main components:

1. LED Control Board
2. LED Platform
3. User Interface

Multiple printed circuit boards (PCBs) were designed for the various sub-systems to modularize the system and facilitate assembly, repair, and reusability. This was achieved in two ways: (1) using commercially available power supply systems and microcontroller boards that are pre-assembled with entire surface mount (SMD) component architecture, and (2) creating custom breakout boards for other electronic sub-systems. These commercially available and custom boards are interconnected through a custom LED control board using header pins and sockets so that they can be easily assembled and replaced in the event of failure, or reconfigured for different LEDs and microplate density. The 96-well version is the subject of most discussion and validation in this publication. However, a 24-well version containing 4 LEDs per well was also designed and tested to allow researchers the ability to irradiate an individual well with more than one wavelength at a time. This also allows for the option of irradiating cells in wells with a larger surface area for downstream experiments (FACS, PCR, western blotting) requiring sufficient cell numbers.

**Fig 1:**
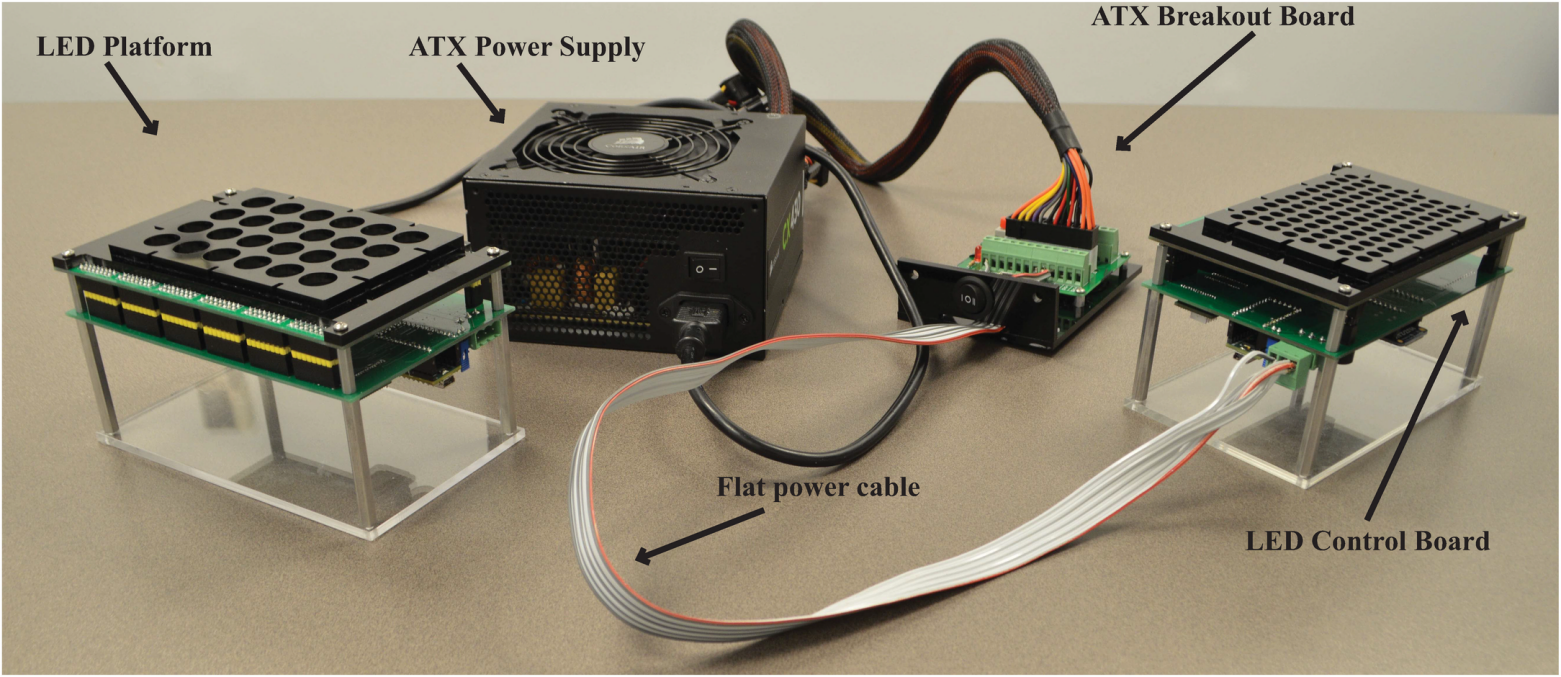
Components of the MPS. Photograph of the MPS, featuring the main device comprising an acrylic base mounting the LED Control Board and LED Platform using aluminum standoffs. The system is powered by standard ATX power supply/ATX breakout board through a pluggable screw terminal with flat cable.

The MPS, shown below in Fig 1 consists of an acrylic base with aluminum standoffs supporting the LED control board and LED platform. The LED platform is mechanically and electrically connected to the LED control board via standard.1” pitch header pin sockets. A black acrylic isolation plate is secured to the board via standoffs, which supports a standard 96-well black-walled microplate for simultaneous, independent administration of light to individual wells (1 LED per well).

### LED control board

The LED Control Board consists of multiple subsystems that together power and control the LEDs on the LED Platform according to commands received through the user interface. The subsystems are:

1. Teensy 2.0 (a breakout board for an ATmega32U4 microcontroller)
2. Adafruit Huzzah ESP8266 breakout (a Wi-Fi Module based on the Expressif ESP8266 system-on chip)
3. Array of LED driver modules (each a breakout board for a Texas Instruments TLC59401)
4. ATX Power Supply (a standard personal computer power supply which delivers high-current 5 or 3.3 V LED power voltages)

**Fig 2:**
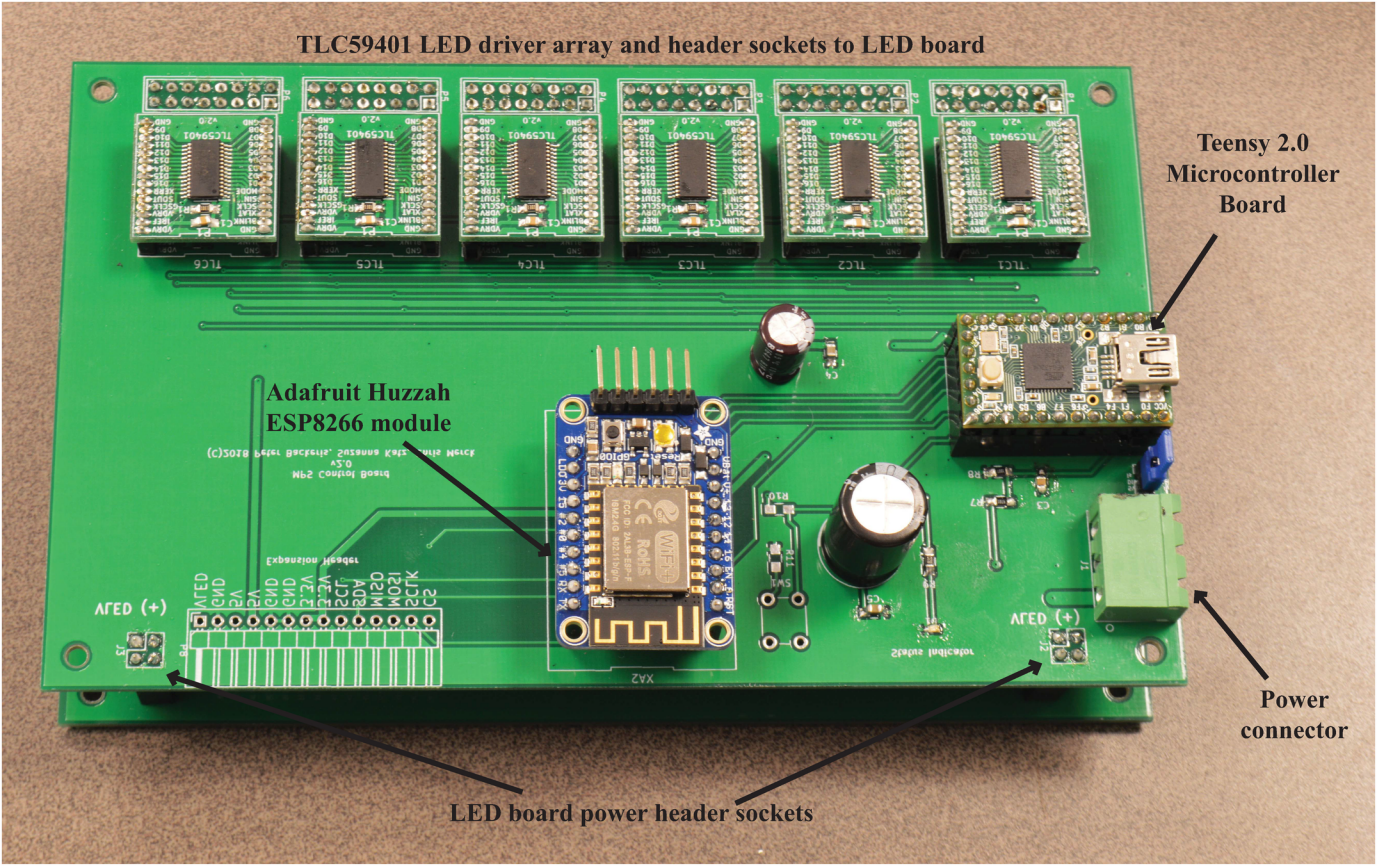
LED control board. Photograph of the MPS LED Control Board, with key components and connectors identified. Only solder points are visible for labeled header sockets as the sockets are on the other side of the board.

These subsystems, with the exception of the power supply, are depicted in Fig 2 below. The power supply is separate from the board and connected to the LED control board through a pluggable screw terminal and 5-wire 18-gauge flat cable Fig 1).

The Teensy 2.0 is a commercially available, open-source, low-cost, pre-assembled breakout board for the Atmel ATmega32U4 microcontroller—an 8-bit, 16 MHz AVR microcontroller with 1 kB EEPROM, 32 kB program memory, and 2.5 kB SRAM. The Teensy 2.0 is Arduino-compatible and custom firmware was written using the Arduino IDE to communicate with the system’s peripherals, receive experimental protocol from user, and control the LED driver array. The firmware features an ASCII-based serial port command interface to control the LEDs and run experimental protocols (described later). This can be controlled via a direct USB serial port connection, or through a wireless connection and web browser graphical user interface (GUI) via the Huzzah ESP8266.

An Adafruit Huzzah ESP8266 Wi-Fi module breakout board was integrated into the LED control board to provide wireless control capabilities through a web browser. The ESP8266 module is connected to the Teensy 2.0’s second serial interface and acts as a bridge for the ASCII-based serial command interface. The Huzzah module acts as a wireless access point and server for a web browser-based graphical user interface (GUI) that relays information from the Teensy microcontroller to the user, as well as relaying commands from the user back to the Teensy (described later).

The LED driver array consists of 6 TLC59401 LED driver integrated circuits (ICs), individually assembled onto a custom breakout board which then connect in place to header sockets on the control board. The TLC59401 is a 16-channel constant-current sink, pulse-width modulation (PWM) LED driver IC. The drivers provide 16 channels of 6-bit (64 level) current and 12-bit (4096 level) PWM control to drive 16 independent LED channels, with a maximum current of up to 120 mA per channel. The driver boards are controlled through a single, daisy-chained serial connection to the Teensy 2.0. A breakout board with 1 mm-pitch header pins was designed to allow for drop-in installation to the control board and easy replacement in the event of failure. Six of these boards are used on the LED Control Board to enable 96 independently controlled LEDs. Small heat sinks were also adhered to the top-surface of the driver IC packages with silicone-based thermal glue to aid in heat dissipation. Additional design details are provided in S1 File.

The driver boards are controlled by the Teensy 2.0 microcontroller board, which in turn control the brightness of LEDs through two parameters: pulse-width modulation (PWM) duty cycle and current. The 12-bit (4096 level) PWM duty cycle value is set for each channel on the driver. PWM by the driver cycles the LED power on and off at a high frequency provided by the microcontroller PWM clock pin, and the PWM duty cycle value defines the portion of the cycle that the LED is on (a value of 0 being always off and 4095 being always on). This has the effect of modulating the average irradiance (units of mW/cm2) of the LED. The 6-bit (64 level) current value, set for each channel as well, controls the instantaneous current amplitude provided to the LED during the channel’s on-period of the PWM cycle. A value of zero provides no current to the LED while a max value of 63 provides the maximum current as set by the external current reference resistor on the breakout board. This hardware-defined maximum current is a global value shared by every channel on the driver board, and can be set to anywhere from 0 to 120 mA according to the specifications of the LEDs used. The combination of grayscale and PWM provides 2-factor control of the irradiance for each LED, with the 6-bit current providing coarse control and 12 bit PWM duty-cycle providing fine adjustment.

A semi-automated calibration system was devised using a commercially available ThorLabs PM100D USB Power Meter and a custom Windows GUI calibration utility to streamline the calibration process, which is explained in detail in the S2 File. This calibration utility allows users to automatically measure irradiance versus current value and fit the measurements to a quadratic formula, upload and store calibration coefficients to the MPS, as well as check the accuracy of any calibrated LED through automated software routines.

An ATX power supply was chosen as the power source due to its low cost, wide availability and high power levels. ATX power supplies are normally found in desktop PCs, connecting to the motherboard and other peripherals, providing ±12V, 5V, and 3.3V power rails with total power typically 400–700 W, more than adequate to power two or more MPSs simultaneously at maximum power. An ATX breakout board connects to the 24-pin ATX connector and provides screw terminals for all power rails in the ATX power supply. A 5-wire 18 gauge flat cable is connected to the ATX breakout board to deliver 3 channels of power to the LED control board: ground, 5V to power control board circuits, and switchable 5V or 3.3V for the LED power. The LED power and ground channels each used 2 of the 5 wires to reduce resistance and voltage drop at high current. The ATX breakout board was mounted to an acrylic base with a switch to select between 5V and 3.3V power for the LEDs, and magnets were attached to the bottom to allow mounting of the breakout board to the door or side of metal incubators.

### LED platform

The LED platform is composed of a separate board designed to support 96 PLCC-2 surface-mount LEDs and an overlaying black isolation plate that sits on top to isolate LED light channels to their corresponding microplate well and prevent parasitic irradiance. PLCC-2 is an industry-standard package size and footprint for LEDs which is widely available, and provides ideal size, power, and wavelength options needed for the vast majority of biological photoirradiation experiments. This LED type also has very good beam homogeneity in comparison to other LED types such as standard through-hole (which tend to have a highly variable and non-uniform beam). This aspect is important to maximizing uniform irradiance of cells within a microplate well. An analysis of the radial homogeneity of LED output is presented in S2 File. Wavelengths are currently available in the 405–960nm range, providing options for near-UV and near-infrared experiments as well as all other spectra in between. The separation of the board from the control board allows for future versions of the board to be made through-hole LED compatible, which—though sub-optimal in performance compared to PLCC-2 LEDs—have an even greater selection of wavelengths available that may be needed for some studies.

**Fig 3:**
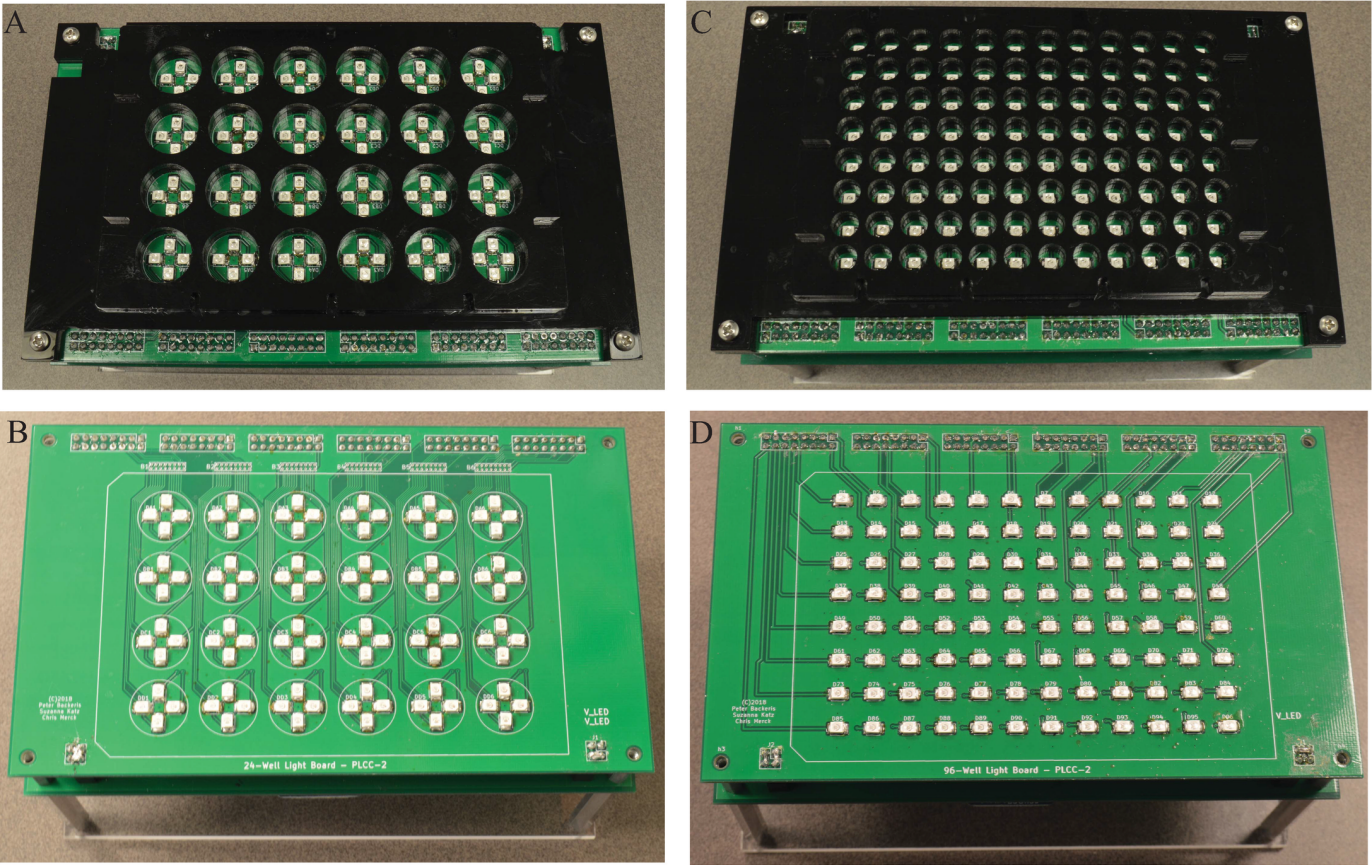
Photographs of the 24-well and 96-well LED platforms and boards. 24-well LED platform with (A) and without (B) isolation plate and 96-well LED platform with (C) and without (D) isolation plate.

The pads on the PCB are positioned to have one LED directly in the center of each well on a standard 96-well microplate, or 4 LEDs spaced evenly around the center of each well in the case of the 24-well version (see Fig 3). Each LED is connected to an independent channel on one of the 6 TLC59401 LED drivers on the LED control board via its corresponding header pin. Six 8x2 2.54mm header sockets on the LED board are aligned with their corresponding sockets on the LED control board, connecting each driver to a section of LEDs on the LED board. An atypical design approach was used to mate the pin headers of the LED control board to the LED board. Rather than using male pins on one board and female sockets on the other, female sockets were soldered on both boards and were mated via extra-long symmetric male header pins. This was done to increase the space between the LED board and the LED control board, helping reduce any potential heating effects, as well as to make accidentally damaged or bent pins inconsequential as they can be readily replaced. This connection scheme mates the boards together both electrically and mechanically, and provides modular flexibility in reconfiguring a control board to connect to interchangeable LED platforms for different sets of experiments. This also allows either board to be replaced while keeping the other (in the event of failure of one of the boards), and reduces potential heat transfer from the driver boards to the light platform (minimizing heat effects on cells).

The LEDs share a common positive voltage power plane, which is connected to the LED Control Board via 2 sets of 4x4 header sockets on the opposite side of the board as the LED driver channel connections. This provides either 5 or 3.3V depending on the specifications of the LEDs. LEDs with a forward voltage of 2.8V or less are given 3.3V power, which reduces the power dissipation and heat generation by the LED driver boards, while LEDs with forward voltage above 2.8V receive 5V power. The user selects the LED voltage using a selector switch on the ATX breakout board base as described previously. It may be desirable to have different wavelengths in different wells on a board, or even within the same well (possible with the 24-well system). In this case, the voltage will need to be set to 5V if any of the LEDs require that voltage, which may be sub-optimal in terms of power dissipation by some of the LED drivers (but would still work). In the case of different LEDs within the same well on the 24-well MPS, these LEDs would have to share the same LED driver and therefore the same maximum current. If one type of LED in the well has a higher maximum current that needs to be attainable for the experiment, the lower maximum current LEDs will need to be limited in software to prevent overdriving the LEDs (by setting a maximum on the 6-bit current value). Otherwise, the external current reference resistor can be set to accommodate the lower maximum-current LED.

An isolation plate was designed in Fusion 360 CAD software to fit over the LED board, align a standard 96 or 24-well black-walled microplate over the board, and isolate the light from each LED to reach only the corresponding well in the microplate. Two black acrylic sheets cut with a laser cutter and solvent bonded together were used to fabricate the isolation plate. The isolation plate is aligned and mounted to the LED board with corner screws that attach to a second row of standoffs stacked top of the LED control board standoffs.

### Firmware and user interface

The Teensy 2.0 firmware, written in C++ in the Arduino integrated development environment (IDE), controls the operation of the LED channels by interpreting ASCII-based commands received through a serial connection to the Huzzah ESP8266 Wi-Fi module, and then updating the channel grayscale (PWM) and dot-correction (current) settings in the TLC59401 LED driver array accordingly. This command interface can be used manually to set LED irradiance values, or automatically by allowing users to upload pre-configured light program files that specify parameters for groups of 1 to 16 LEDs on a time schedule. This latter mode is the method by which photoirradiation experiments are conducted, as pre-programmed LED parameter groups and timings are sent to the Teensy and stored in memory, and used to control the groups of LEDs using the internal timers (with better than 1ms precision).

The Huzzah ESP8266 Wi-Fi module acts a communication bridge between the user and the Teensy microcontroller, through a serial connection with the Teensy, and Wi-Fi connection with the user’s computer. The Teensy is configured to act as wireless access point, with an SSID that indicates the MPS configuration (wavelength and number of wells + unique identifier), so that the user can connect directly to the MPS through a Wi-Fi connection. Once connected, the user is able to enter a URL in their browser that loads a web page from a webserver running on the ESP8266 Wi-Fi module. This web page is a GUI written in HTML5 and JavaScript that allows the user to control the MPS by sending direct ASCII commands through a text input field on the webpage, as well as by uploading JSON-format light program files containing timing and irradiance parameters for groups of LED channels. These JSON files are generated through a separate browser-based HTML5 webpage GUI, loaded from a file on the user’s computer (this webpage can also be hosted on a web server or the Huzzah Wi-Fi module). This GUI provides a visual diagram with the labeled wells in a 96-well plate, which can be selected, adding them to the current group. Each group is represented by a line in a dynamic table that contains these selected channel numbers (1-96) listed for the group, along with the additional parameters needed for the light program, including:

- Condition name for the group
- Irradiance intensity (in hundredths of mW/cm^2^)
- Duration (s)
- Start Delay (s)
- Number of cycles
- Off time (s)

**Fig 4:**
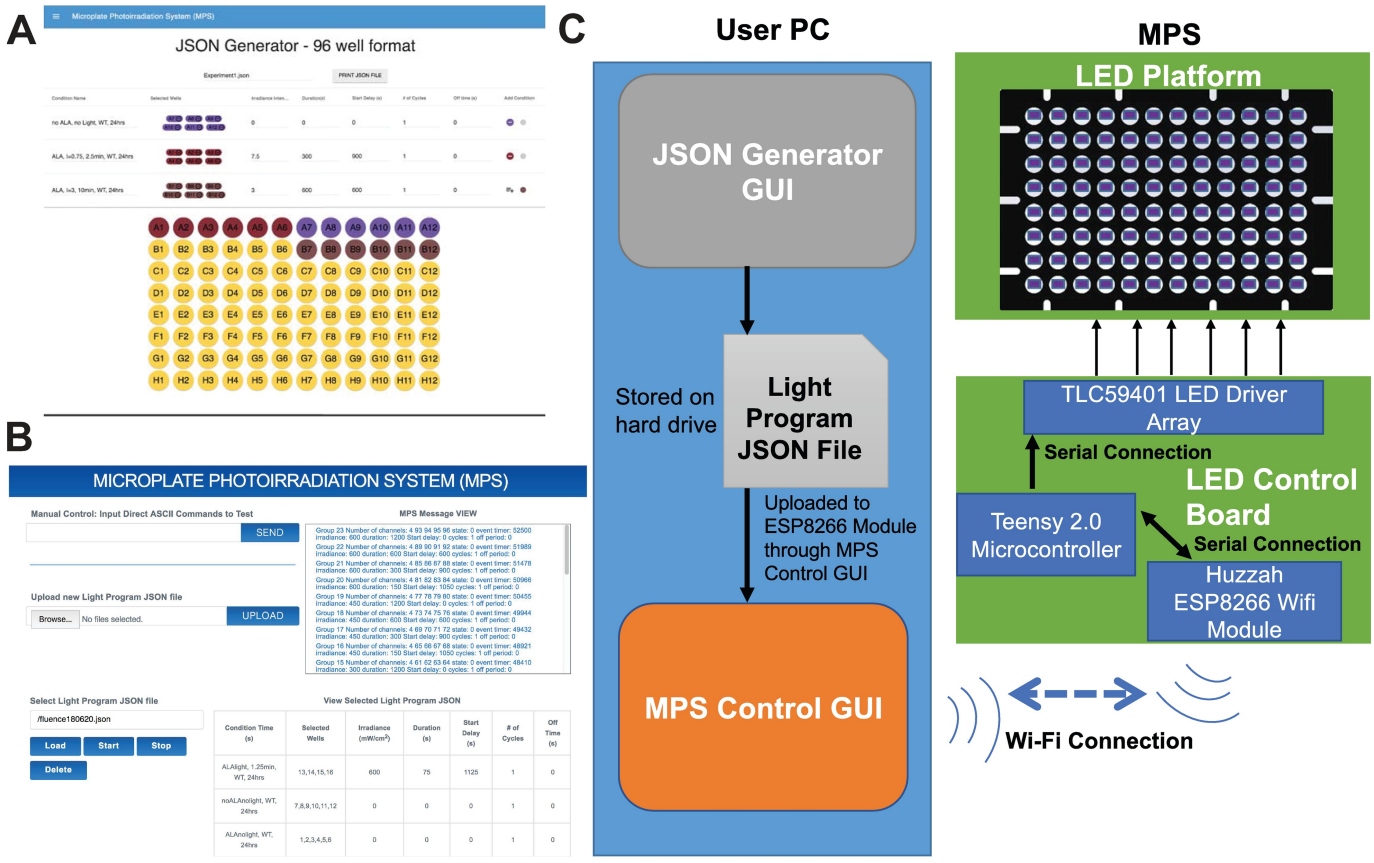
Screenshots of MPS software GUIs and MPS functional diagram. (A) Screenshot of HTML5-based GUI for generating light program JSON files to be uploaded to MPS (B) Screenshot of MPS Control GUI, which is loaded from the ESP8266 Wi-Fi module webserver and allows user to control MPS and upload and run light programs (C) Functional block diagram of the MPS software components, with all major interconnections between software and hardware components shown.

The GUIs for generating light program JSON files, the MPS Control GUI, and a functional diagram of the interconnections of the major software and hardware components of the MPS are shown in Fig 4.

All the channels in the group will behave identically according to these settings. Condition name is just a label for identifying the group (potentially based on other treatment conditions and cell type information) to aid in data analysis at a later point. Irradiance sets the desired power for the channel in mW/cm^2^, which is necessarily a unique pair of PWM and current values for each channel based on calibration parameters for that individual LED (see S2 File). Duration is the time in seconds that the LEDs remain at the set irradiance. Start delay is the time after the start of the light program at which the LEDs start their first cycle, which is used to stagger start times of the groups if desired. Number of cycles is used to set pulsed or repeating cycles of light. A value of 1 is default and lights will only turn on for 1 course of the set duration. Any value greater than 1, will cause the channels to repeat the same irradiance/duration cycle for the number of times specified by this value. Between each cycle, the group will remain off for an interval set by the off time parameter.

Once all of the group settings have been defined in the HTML table, a button in the JSON-generator GUI can be pressed that automatically generates a JSON light program file that is saved to the user’s hard drive. These are the files that can be uploaded to the Huzzah ESP8266 Wi-Fi module from MPS Control GUI as mentioned previously. The JSON files are stored in the flash memory on the Wi-Fi module, and will remain in storage even when power-cycling the system. All JSON light program files stored on the Wi-Fi module are displayed in a drop-down list; the user to select any of them to be run or removed from memory. A detailed comparison of our MPS with recently published open-source photoirradiation devices, along with how these open-source devices compare against a commercially available closed-source system, is presented in S1 Table. A repository containing all source files for the hardware and software is located at https://osf.io/9enpx/.

## Experimental methods and materials

### Cell culture

Human Dermal Fibroblasts (HDFn) were purchased from ThermoFisher Scientific (catalog number C0045C0) were maintained in 10-cm tissue culture dishes with.1% gelatin coating and split 2-3 times per week. Media was composed of DMEM with 15% Fetal Bovine Serum, 1% L-glutamax, 4.5g/L D-glucose, and 1% Pen Strep. For experiments, cells were split and plated unto 24 or 96-well black tissue-culture treated microplates coated with gelatin (“Krystal” black clear bottom 24-well purchased from Denville Scientific (P9835) and black clear bottom 96-well microplates were purchased from Fisher Scientific (Corning™ 3603). After 95% confluence, ALA at 1mM was added into culture media and incubated for 6, 24, or 36 hours.

### Reagents

ALA (5-Aminolevulinic acid) was purchased from Sigma as a 500mg powder and diluted in H20 to a 500mM stock solution and stored in the dark at 4 degrees C. Before each experiment, a fresh working solution was prepared by adding 2uL per 1mL of culture media, yielding a 1mM working solution in which cells were incubated.

### Light treatment of cells

Cell media with ALA was washed off 3x and replaced with 100ul FluoroBrite DMEM in each well followed immediately by a pre-treatment microplate measurement to establish baseline PpIX fluorescence in each well. The plate was then placed on top of the LED platform of the MPS and light treatment was conducted inside the incubator, with a black acrylic plate covering the lid of the plate to block otherwise reflected light from the incubator shelves and walls. The flat power cable is impinged by the gasket of the inner door of the incubator and the connected ATX power breakout board is magnetically mounted to the outside of the main door near the hinge (to reduce range of motion). A JSON file defining the plate configuration for the LED parameters was created using the JSON Generator program and uploaded to the MPS using the web-based MPS Control GUI over Wi-Fi prior to the experiment. The JSON file was selected, loaded and initiated using the MPS Control GUI, beginning the light treatment program.

### Fluorescence assay

The fluorescence was measured by an EnSpire 2100 Microplate reader (PerkinElmer) using excitation/emission wavelengths of 406/635 nm. Porphyrins are a class of tetrapyrrole molecules that are strongly excited by light wavelengths in the Soret band (400-410nm), and PpIX has been shown to have maximal absorbance at 405–407nm [38–40]. The decision to utilize 635nm emission to determine relative PpIX fluorescence was informed by spectral investigations of ALA-induced PpIX detection in cell suspensions [40], in vitro [39], as well as in vivo detection in animal models [41] and humans [42, 43]. An area scan was performed in the center of each well with 12 scans per well (100 flashes per scan). Measurements were taken before and after light treatment allowing calculation of percent loss of fluorescence per well to help normalize data to pre-treatment variability in initial measured PpIX fluorescence. A comma-separated values (csv) file containing a list of measurements for each of the 12 area scans in the well was then exported for analysis.

### Analysis

Data analysis was performed using R/RStudio scripts which were written to import and merge JSON data containing experimental and light treatment conditions with the measurement data contained in the associated microplate assay csv file. The modified Thompson-Tau outlier removal algorithm was employed in the software to remove extreme scans (alpha, 0.01) in the pre-light treatment measurement in each well that would be associated with non-biological interference in the read, such as particles fluorescing or blocking true fluorescence. Corresponding scans were removed the post-treatment measurement data. Scans were then averaged for each well, and outlier removal was applied again (alpha, 0.02) to the pre-treatment data using the Thompson-Tau to remove outlier wells from the analysis that may have had technical issues due to errors in ALA addition, cell confluence, etc. Wells that had 4 or more scans removed during the previous outlier removal step were also removed from the analysis. Corresponding wells in the post-treatment data were then removed from analysis. Percent loss of fluorescence after light treatment was calculated for each well using the average fluorescence for the pre and post-treatment measurement. The JSON files used to control the MPS for the experiment were used by the analysis script to calculate averages and standard deviations for each group of replicate wells. Condition averages and standard deviations for selectable sets of measurements are plotted as either line or bar graphs as shown in the Results section. Hypothesis testing was done using one-way ANOVA with Tukey’s post-hoc adjustment. For all the analyses, p < 0.05 was considered statistically significant.

## Experimental results

### A microplate fluorescence protocol to rapidly detect ALA-induced PpIX-associated fluorescence photobleaching

**Fig 5:**
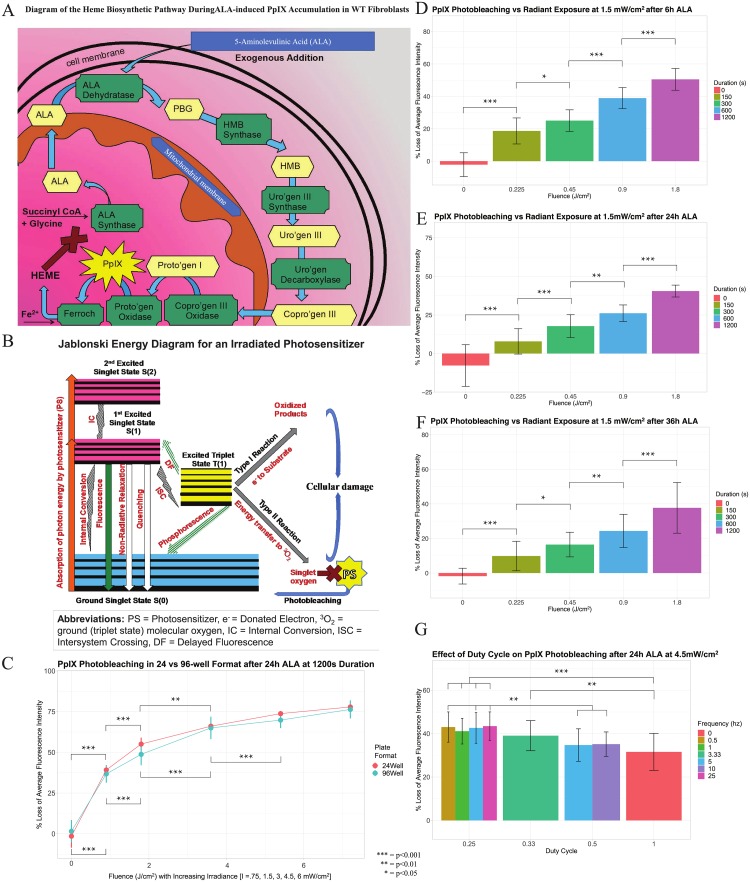
Biological validation of the Microplate Photoirradiation System (MPS) and a microplate fluorescence assay to detect ALA-induced PpIX photobleaching in live adherent wildtype (WT) fibroblasts. (A) A diagram of the heme biosynthesis pathway and PpIX accumulation in an in vitro model of exogenous ALA addition. Abbreviations: ALA = Aminolevulinic acid, PpIX = Protoporphyrin IX, PBG = Porphobilinogen, HMB = Hydroxymethylbilane, Uro’gen = Uroporphyrinogen, Copro’gen = Coproporphyrinogen, Proto’gen = Protoporphyrinogen, Ferroch = Ferrochelatase (B) Biophysical rationale for photobleaching of PpIX, a type II photosensitizer. (C) Experimental validation of PpIX accumulation and photobleaching in 24 and 96-well microplates irradiated with their respective MPS LED boards at fixed duration of 20 minutes and increasing irradiances of 0.75, 1.5, 3, 4.5, and 6 mW/cm2 (resulting fluence values of 0.9, 1.8, 3.6, 5.4, and 7.2 J/cm^2^, respectively). Experimental validation of the photobleaching effect at 6h (D), 24h (E) and 36h (F) ALA incubations using 96-well LED board and microplate to treat cells with 405nm light at a fixed irradiance of 1.5mW/cm^2^ with increasing durations of 150, 300, 600, and 1200 seconds (resulting in fluence values of 0.1, 0.2, 0.5, 0.9, and 1.8 J/cm^2^, respectively). (E) WT fibroblasts treated with a fixed irradiance of 4.5mW/cm2^2^ and fixed fluence of 1.35 J/cm^2^, while varying duty-cycle (25%, 33.3%, 50%, or 100%) and frequencies (0.5, 1, 3.33, 5, 10, 25 Hz). All conditions include data from 3 or more independent experiments (microplate batches) with 4 or more wells per condition in 96-well plates and 3 or more wells per condition in the 24-well plate (before outlier removal). Visual layout of the microplate and the plate configuration for the experimental parameters used in the light treatment, as well as plate ID’s of the microplate batches used in the experiments, are outlined in S3 File. Details regarding the number of samples used in each experimental condition, the means and SDs for each condition used to plot the data on the graphs, and the respective microplate batches utilized for each graph, are presented in S1 Data.

In mammalian heme-producing cells (bone marrow and fetal liver) the condensation of Succinyl-CoA and glycine by the enzyme 5-aminolevulinic acid synthase produces 5-aminolevulinic acid (ALA), which commits the cells to continue onward in the production of heme [9, 44]. Eight ALA molecules are needed to eventually form one PpIX molecule; the enzyme ferrochelatase then incorporates an iron molecule into the center of the tetrapyrrole to produce heme. Heme biosynthesis can be induced even in cells that do not naturally produce heme, as shown in the heme pathway diagram, Fig 5A. In this model, PpIX accumulates temporarily in the mitochondria due to the inability of ferrochelatase enzyme to efficiently convert it to heme (see Fig 5A legend for abbreviations). The accumulated intermediate causes the cell to be photosensitized, as it is able to absorb light and produce biological changes due to absorption of photons. The path of an absorbed photon by a photosensitizer like PpIX begins at the ground state, leads to a jump to the singlet excited state(s) and emits fluorescence as it returns back to its ground state (like other fluorophores). However, it can alternatively proceed to the longer-lived excited triplet state through a process called *Intersystem Crossing*, a critical feature of most potent photosensitizers and of tetrapyrroles in particular [45–47]. From there, the photosensitizer is positioned to directly transfer energy to molecular oxygen (a triplet in its ground state) which converts it to cytotoxic singlet oxygen in a Type II reaction, Fig 5B. Type I reactions which involve electron transfer to produce oxygen radicals are also possible for triplet state photosensitizers, though it has been well established that photosensitization by PpIX proceeds primarily through a Type II mechanism [46]. The type II reaction—in part due to the low energy gap between this tetrapyrrole and molecular oxygen and in part due to the short diffusion distance of the cytotoxic singlet oxygen [47]—is the reason why PpIX photosensitization can be utilized in the destruction of malignant and aberrant tissue types with ALA-based PDT. Since the singlet oxygen is thought to be the primary species produced during a type II reaction, it may sometimes interact with the photosensitizer itself and destroy it in a process known as *photobleaching*. The singlet oxygen-mediated damage applied to the cells, termed *the photodynamic effect*, can thus be estimated by measuring photobleaching kinetics [46, 48]. This protocol—called *implicit dosimetry*—has been applied in both in vitro and in animal models [46, 48–51]. Here, we exploit the ALA-induced PpIX accumulation and fluorescence photobleaching model to demonstrate the utility and reliability of the MPS for high-throughput photodynamic therapy research. Our protocol measures PpIX-associated fluorescence in live, adherent fibroblasts after various ALA incubations.

Fig 5C shows WT Fibroblasts were incubated with ALA for 24 hours in either a 24-well or a 96-well microplate for 24 hours; 405nm light was delivered (inside the incubator) with the 24 or 96-well MPS, respectively. The duration was kept fixed at 20 minutes while the irradiance was varied (0.75, 1.5, 3, 4.5, and 6 mW/cm^2^) between conditions. Thus, fluence was modulated with the irradiance parameter (since *fluence* = *irradiance* ⋅ *time*) and the percent of PpIX photobleaching is depicted with increasing fluence along the x-axis. For both the 96 and 24-well formats, there is significant photobleaching observed (p < 0.001) between no light and 0.75mW/cm2^2^ irradiance (a fluence dose of 0.9 J/cm2), as well as between the.75mW/cm2^2^ and the 1.5mW/cm2^2^ condition (a fluence dose of 1.8J/cm2^2^). There is also significant difference between the 1.5mW/cm2^2^ and 3mW/cm2^2^(fluence dose of 3.6J/cm2^2^) conditions in both the 96-well(p < 0.001) and 24-well (p < 0.01) formats. The difference between light treatment at an irradiance of 3mW/cm2 and 4.5mW/cm2 (a fluence dose of 5.4J/cm2) was significantly different for the 96-well formats but not for the 24-well formats (even though the 2 microplate formats did not have values significantly from each other at that point). This is likely due to the smaller number of samples per plate used for the 24-well experiments (3 independent plates with 3 wells per plate in the 24-well and 3 independent plates with 7 wells per plate for the 96-well). There was no statistical significance between light treatment of irradiance 4.5mW/cm2^2^ and an irradiance of 6mW/cm2^2^(a fluence dose of 7.2J/cm2) in either microplate formats. Importantly, there was no significant difference in photobleaching between the 2 microplate formats at all values of delivered fluence.

The next experiment involved ALA-incubated cells being treated with increasing duration of 405nm light at a fixed irradiance of 1.5mW/cm^2^. The PpIX-associated fluorescence photobleaching at 6h (Fig 5D), 24h (Fig 5E), and 36h (Fig 5F) ALA incubation time-points was assessed, with every duration of radiant exposure (5, 10, and 20 minutes) having statistically significant differences between each condition within the incubation timepoint and also relative to ALA – no light control. The difference was less pronounced, though still significant (p < 0.05), between the 2.5 minutes (fluence dose of 0.2J/cm2^2^) and 3 minutes (a fluence dose of 0.5J/cm2^2^) durations in the 6h and 36h ALA incubation graphs. The difference was also less pronounced in the 24h and 36h ALA incubations between the 3min duration and the 10 minute durations, though also still significant at p < 0.01. All other interactions were significant at (p < 0.001), with the full list of interactions and p-values presented S1 Data.

In Fig 5G, the effect of pulsation on PpIX photobleaching was explored at a fixed fluence of 1.35 J/cm^2^ and varying duty-cycle (25%, 33.3%, 50%, or 100%) and frequencies (0.5, 1, 3.33, 5, 10, 25 Hz). There is a significant increase in the percent of photobleaching between continuous light (duty cycle of 1) and when when light is pulsed at duty cycles of 33.3% ((p < 0.01) or 25% (p < 0.001). There is also a significant difference in photobleaching between light that is pulsed at 50% and 25% duty cycle. There was no significant difference in PpIX photobleaching levels where frequency conditions were varied but where duty cycle was kept constant.

### The effect of fluence on the photobleaching kinetics of PpIX-associated fluorescence

**Fig 6:**
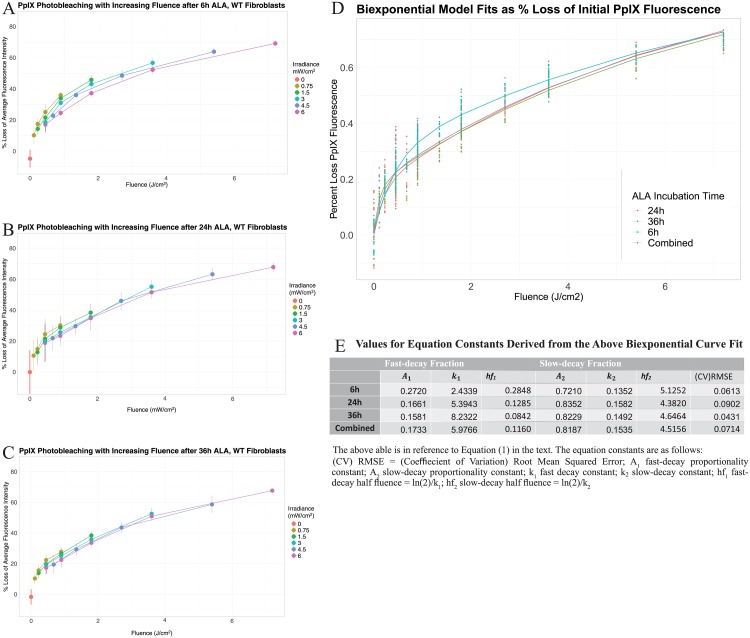
Photobleaching of PpIX in live adherent wildtype fibroblasts as a function of 405nm radiant exposure. (A, B, C) Comparison of the PpIX photobleaching kinetics as a function of fluence for each irradiance condition, for the 6h, 24h, and 36h ALA Incubation time-points. (D) Biexponential decay model fitted to PpIX photobleaching, represented as percent loss in fluorescence from initial value as function of delivered fluence (points = raw data, lines = fitted model) for 6, 24 and 36h ALA incubations as well as the model fitted to the combined data. (E) Coefficients for biexponential fit of data in the previous graph, according to the equation described in the text. The “half-fluence”, denoted as *hf*, is a transformation of the exponential decay constant to represent the fluence required to reduce fluorescence by half (analog of half-life). All conditions include data from 3 or more independent experiments (microplate batches) with 4 or more wells per condition in 96-well plates (before outlier removal). Visual layout of the microplate and the plate configuration for the experimental parameters used in the light treatment, as well as plate ID’s of the microplate batches used in the experiments, are outlined in S3 File. Details regarding the number of samples used in each experimental condition, the means and SDs for each condition used to plot the data on the graphs, and and the respective microplate batches utilized for each graph, are presented in S1 Data.

To explore ALA-induced PpIX photobleaching kinetics as a function of fluence, a variety of irradiances and radiant exposure durations were set up on a single 96-well microplate, with ALA added to all conditions except the no ALA – no light control used for background subtraction. In addition to the ALA – no light condition, combinations of 5 positive values of irradiance and 5 positive values of duration created a total of 24 distinct groups with any of 12 possible total fluence values (see S3 File for details on these conditions). The effect of these delivered on PpIX fluorescence with the combination of different irradiances and durations of treatment were then plotted to compare the 6h, 24h, and 36h ALA incubation experiments (Fig 6A, 6B and 6C, respectively).

Next, the PpIX photobleaching kinetics as a function of initial fluorescence and fluence were modeled by a biexponential decay equation:

$$\begin{array}{c} F=f_{i}\left(A_{1}e^{-k_{1}x}+A_{2}e^{-k_{2}x}\right) \end{array}$$ (1)

Where *F* is the PpIX fluorescence (background-corrected AFI) after irradiation, *f*_*i*_ is the initial PpIX fluorescence, *x* is the radiant exposure or delivered fluence (J/cm^2^), *A*_1_ is the amplitude coefficient (fraction) of a fast-decay component, *k*_1_ is the fast decay constant (cm^2^/J), *A*_2_ is the amplitude coefficient (fraction) of a slow-decay component and *k*_2_ is the slow decay constant.

The models for 24h, 36h, 6h ALA incubation times as well as the set of combined data points were fit (Fig 6D using nonlinear least squares regression in R-Studio and the values for model coefficients are given in the table in Fig 6E. The accuracy of the fitted model was assessed by calculating the coefficient of variation of root mean squared error (CV)RMSE of the residuals, which provides a relative standard of error of the residuals normalized to the mean of the data. All (CV)RMSE were less than 0.10, meaning the RMSE value was less than 10 percent of the mean of the data for each model, with the (CV)RMSE being as low as.0431 for the 36h fit.

The decay constants, *k*_1_ and *k*_2_ were transformed into half-fluence values, *hf*, by dividing *ln*(2) by these values. This provides a more intuitive means of comparing the decay constants by indicating the fluence required to reduce fluorescence by half for the slow and fast-decaying components. The *hf* values reveal increasing rate of decay from 6h to 36h ALA incubations for the fast-decay component, with a higher fraction *A*_1_ of fast-decay component in the 6h ALA incubation. The slow-decay component fractions and decay constants were similar for all ALA incubation times. The combined data also produced an accurate model, with a (CV)RMSE value of 0.0714. The amplitude coefficients approximate that about 17 percent of the photobleaching can be accounted for by the fast-decaying fraction of PpIX (half fluence of 0.12 J/cm^2^) and 82 percent by the slow-decaying fraction (4.5 J/cm^2^).

## Discussion

A novel microplate photoirradiation system was designed, fabricated, and validated using a microplate fluorescence protocol that effectively assays photobleaching kinetics of PpIX in live adherent cells.

### PpIX Photobleaching Assay in live adherent fibroblasts

A major practical and regulatory roadblock to mainstream PDT use is the ability to effectively determine the appropriate dosages for specific cancer types and individual patients; these parameters can differ in terms of wavelength needed to penetrate deep enough to reach the tumor (if not skin related), the tissue oxygenation levels of the irradiated area, an individual’s endogenous porphyrin production, and the dose needed to kill cells of a specific cancer type while minimizing damage to nearby healthy cells. For this reason, PDT dosimetry is an active area of research and high-throughput and standardized methods are needed to quickly determine doses that will be effective in a given patient.

The production of PpIX is a photosensitization model currently utilized in clinical PDT, often involving the exogenous addition of ALA which commits the cell to proceed with enzymatic conversions eventually leading to PpIX accumulation in the mitochondria. Protocols developed in the 1950s and 1960s in regards to the extraction and calculation of the photosensitive porphyrins rely on cell lysis and extraction of the specific porphyrin isomers with acids in order to separate the product I and product III isomers and their respective coproporphyrin derivatives [52]. The porphyrin mixtures can be separated with combinations of acid and chromatography techniques; they can then assayed for the distinct emissions with a fluorometry or spectrofluorometry. These methods do not recover the species entirely and the recovery yield tends to differ for the specific porphyrin isomers; the extraction techniques also expose the short-lived metabolites to variables that may affect the ultimate readout as well as creating opportunities for human error. For example, porphyrins can rapidly oxidize in response to pH, oxidizing agents, air, and especially light. The extraction process additionally prevents the assaying of the immediate effects of porphyrins on their cellular environment, including those that are released into the media following specific treatments like photoirradiation. Thus, we explored whether PpIX fluorescence and photobleaching can be detected in live adherent fibroblasts immediately after irradiation and in the same assay media used during the light treatment. We used an area scan feature of the microplate reader to account for the cells being adherent and to exclude fluorescence at the very edges of the well. We detect PpIX by its distinct excitation/emission spectra without first extracting it from the cells, and thus the assay is substantially quicker, simpler, and cheaper.

### Experimental validation of photobleaching assay and MPS

We demonstrate that our assay can effectively detect the percentage of PpIX photobleaching occuring in WT fibroblasts incubated with ALA and that this assay can be done comparably in either the 24 or 96-well microplate formats. When these ALA-photosensitized fibroblasts are treated with either increasing levels of irradiance (0.75, 1.5, 3, 4.5, 6mW/cm^2^) or increasing durations of 405mn light (0, 5, 10, 20 minute exposures at 1.5mW/cm^2^) there is a fluence-dependent increase in the percentage of photobleaching that occurs at every ALA incubation time tested (6h, 24h, or 36h). However, when fluence and irradiance are kept constant (at 1.35J/cm^2^ and 1.5mW/cm2, respectively), we show that decreasing the duty cycle can have a significant effect on the amount of photobleaching that occurs whereas frequency was not a significant factor impacting the percent of PpIX photobleaching when duty cycle was kept constant. This finding is corroborated by a number of in vivo studies in animals [53, 54] and humans [55] looking at the effect of fractionated versus continuous light output on photobleaching in PDT, which observed faster photobleaching when light was fractionated (pulsed). This effect has been shown to be related to oxygen consumption and diffusion kinetics, as photobleaching of PpIX is oxygen and light dependent [54–56]. The existence of a dark period in pulsed light allows oxygen stores to replenish which increases the efficiency of photobleaching, and the same total fluence as continuous irradiance will result in a greater photobleaching effect [53–56].

A biexponential decay model was used to model the photobleaching kinetics because the monoexponential equation exhibited poor fitting by failing to model the faster initial rate of decay observed at all ALA incubation times. The biexponential model, which is the sum of two independent first-order exponential decays, better captures the presence of the fast and slow decay components seen in the graphs. The solutions to the model fitting reveal the first exponential component accounting for the fast initial rate of decay, while the second exponential component accounts for the latter slower rate of decay. The same biexponential decay was observed by Sudworth et al. [57] when monitoring PpIX fluorescence photobleaching dynamics at micrometer scale locations in formalin-fixed keratinocytes and fibroblasts, though in this case irradiation was performed with 532nm light. Moan et al. [58] showed similar biexponential decay when investigating PpIX accumulation in WiDr cells (derived from a primary human adenocarcinoma of the rectosigmoid colon) and theorized that this could be due to PpIX molecules moving to different binding sites during irradiation. They suggested that the biexponential kinetics observed can be accounted for by the degradation of PpIX in two different environments—that which is bound and that which is not bound to proteins [58]. Robinson et al. [50] also showed that second-order rather than a first order model was a better fit in the ALA-induced PpIX photobleaching occurring in the hairless skin of a SKH HR1 mouse, while the findings of Georgakoudi et al. [59] supported the notion that simple fluence-based mono-exponential decay kinetics could not accurately account for the photobleaching kinetics of PpIX. They additionally emphasized the correlation these photobleaching kinetics had to the photodamage effect and the importance of understanding the proper mechanism of sensitizer photoinactivation in order to predict the temporal and spatial distribution of singlet oxygen. These investigations corroborate our own findings and underline the importance of studying photobleaching kinetics in furthering the translational potential of Photodynamic Therapy.

The combination of the microplate fluorescence assay developed here along with the capabilities of the MPS would allow for further photobleaching investigations using various wavelengths of light, pulsed irradiance parameters, other photosensitizers and additional compounds that may modulate photobleaching kinetics.

### Conclusion

The validation of the MPS with the ALA-PDT model demonstrate the utility of a highly controllable, configurable, and precise photoirradiation system for studying the effects of irradiance and fluence of a particular spectra of light on adherent cells. The importance of criteria in the design of this system (discussed in the introduction) were evident in the course of performing the experiments and analyzing the results. For example, the combination of calibrated irradiance for each individual well, and flexibility in configuring independent irradiance conditions on an individual microplate basis, allows for relatively small numbers of experiments to be conducted to see consistent effects with high statistical significance. This can be particularly valuable when precious cell samples are being studied, or when challenging and expensive experimental procedures, assays, or reagents are involved upstream or downstream of the photoirradiation experiments. The user-friendly software, wireless control, and modular design make the system more convenient to use, reconfigure and maintain.

With the ability to calibrate and standardize irradiation parameters, the 24-well MPS can be used in conjunction to the 96-well MPS when a large number of cells are needed for specific conditions, or when morphology and cellular localization needs to be photographed during microscopy. The 24-well format MPS containing 4 LEDs per well can also be useful for probing the biological effects of wavelength combinations (such as those required in optogenetics or photobiomodulation experiments). For example, it has been demonstrated that the combination of blue and red light irradiation has unique effects on skin cells [60]; this type of biological phenomenon could be easily studied by soldering two or more different wavelength LED’s onto a 24-well LED board. Either the 24 or the 96-well boards can also be soldered with different types of LED in one single board, allowing for different wavelengths of light to be tested in a single experiment.

To our knowledge, this is the first photoirradiation system that can deliver individual well control in a 96-well microplate (either open-source or commercial), a frequently used format in high-throughput experiments. It is also the first open-source system that allows for interchangeable 24 and 96-well formats, surface mount LEDs (that tend to deliver more homegenous light than through-hole versions), and as many as 4 LED’s per well in the 24-well format. The only other currently available microplate photoirradiation system that has surface mount LEDs and as many as 4 LED’s per well is the commercial LUMUS system, described by Clements et al. [61], a device which costs thousands of dollars and which only irradiates at 4 fixed wavelengths of light (though does have its own built-in temperature control and can handle both 24 and 48-well formats. Our MPS is also the first photoirradiation system (open-source or commercial) that allows for Wi-Fi enabled control. These novel features are further highlighted in the Light System Comparison Table in S1 Table.

The modular nature of the presented protocol and irradiation system allows for a streamlined user experience and the ability to customize and modify every step of the experimental process. Thus, in addition to ALA-PDT photosensitization, the MPS can be used in a multitude of high-throughput photobiology, photobiomodulation, photochemistry, and optogenetic experiments.

## Supporting information

S1 File Design and fabrication of the MPS. (PDF)

S2 File LED control and calibration. (PDF)

S1 Table Light system comparison chart. (PDF)

S3 File Plate configurations and experimental parameters. (PDF)

S1 Data Data and statistics for Figs 5 and 6 graphs. (XLSX)
