# Supplementary material for: Design and validation of an open-source modular Microplate Photoirradiation System for high-throughput photobiology experiments
Source: PLoS One. 2018 Oct 5;13(10):e0203597. doi: 10.1371/journal.pone.0203597 (PMC6173374; doi:10.1371/journal.pone.0203597)
Supplement: S1 File — (PDF) [file pone.0203597.s001.pdf]

S1. MPS Design and Fabrication

Printed Circuit Board Design and Fabrication

The printed circuit boards (PCBs) for the LED control board, LED boards, and TLC59401 LED driver breakout boards were designed in KiCAD and fabricated using GoldPhoenixPCB (Wuhan, China). Circuit boards were assembled in-house using hot plate with hot air for the LEDS and manual SMT soldering techniques for other components as follows. For the soldering of LEDs to the LED boards, a laboratory hot-plate supporting temperatures up to 300C was set to 155 C, and the PCB was placed on the hot plate surface with the LED side facing up, and a chisel-tip soldering iron was used to tin all LED pads with 62/36/2 Sn-Pb-Ag solder. Solder flux was applied using a syringe to each LED pad, followed by placing LEDs as centered as possible on each pad. A hot-air tool set to 330C was used to apply hot air sequentially to each LED pad until the solder melts and the LED settles and centered on the pad. The hot plate maintained the PCB at a working temperature, minimizing the time required with the hot-air tool to melt the solder and reducing risk of burning LEDs from the hot air. The hot air method melted both the anode and cathode joints of the LED simultaneously, allowing the LED to self-center on the pads, which was important to ensure alignment with the center of each microplate well. All other through-hole components including connectors, headers, and passive components were soldered using standard soldering iron techniques.

Schematic Diagram of the LED Control Board designed in KiCAD PCB design software

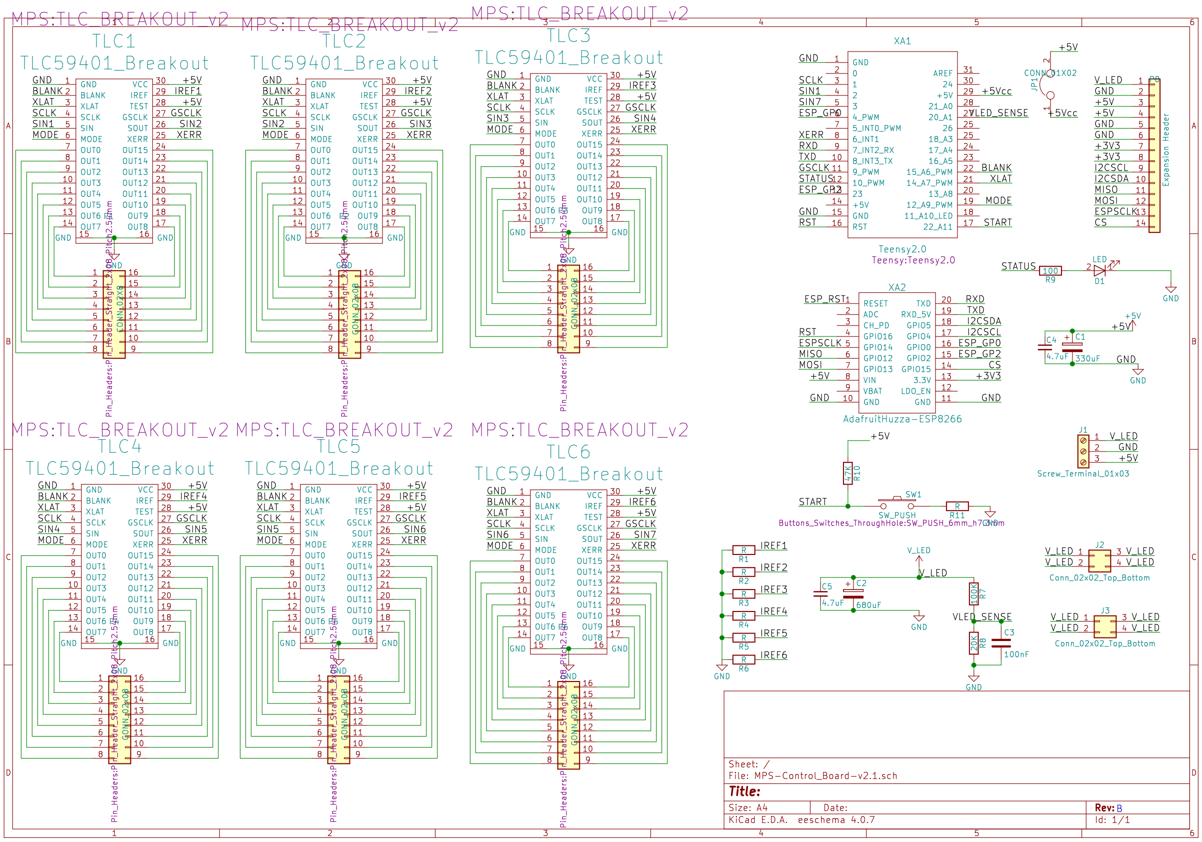

LED Control Board PCB designed in KiCAD PCB Design Software

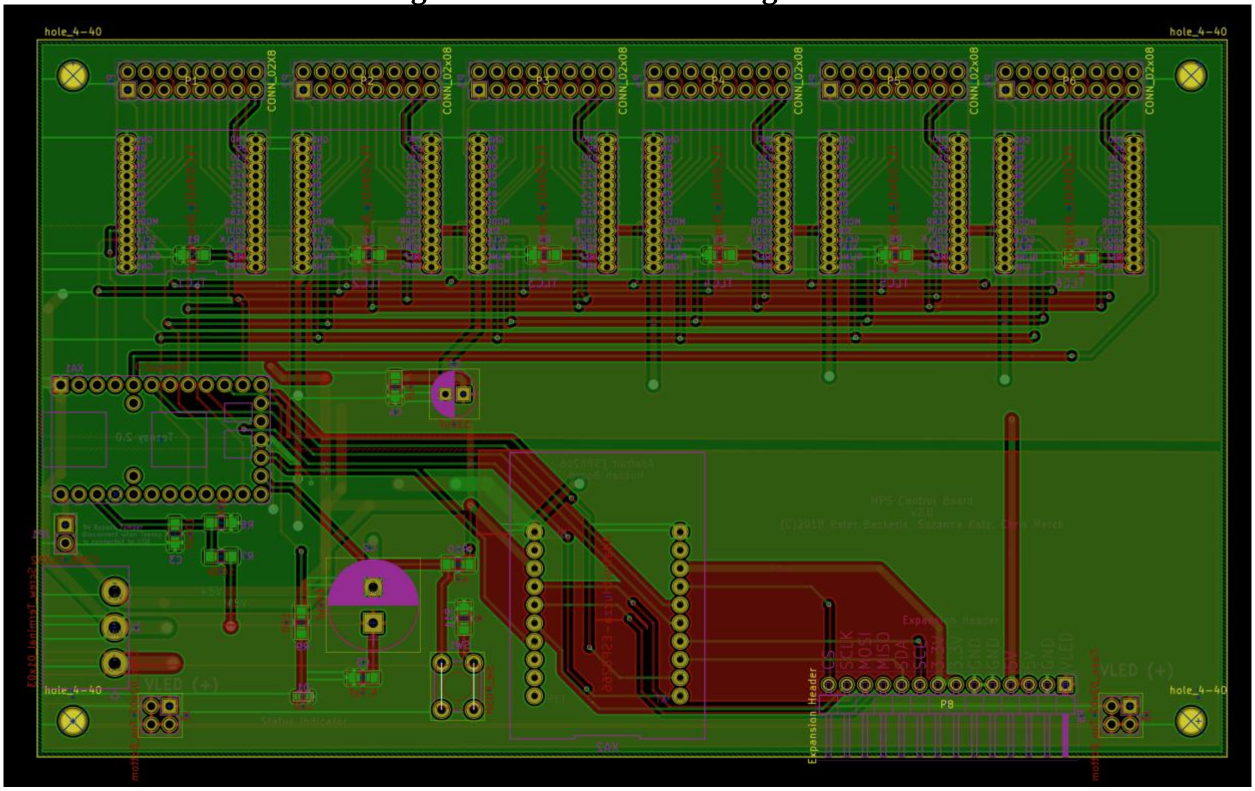

TLC59401 Breakout Board Designed in KiCAD

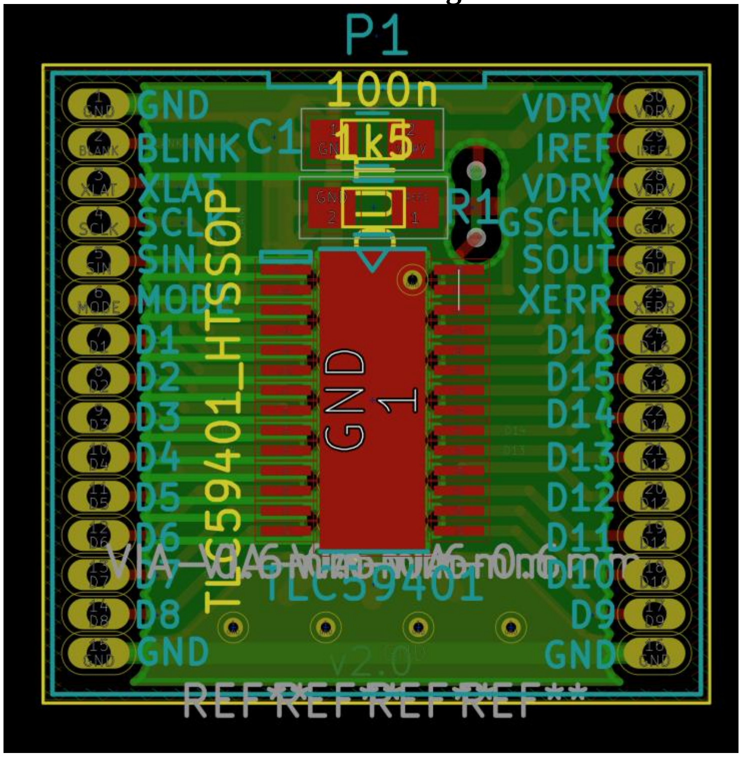

96-Well LED Board PCB designed in KiCAD

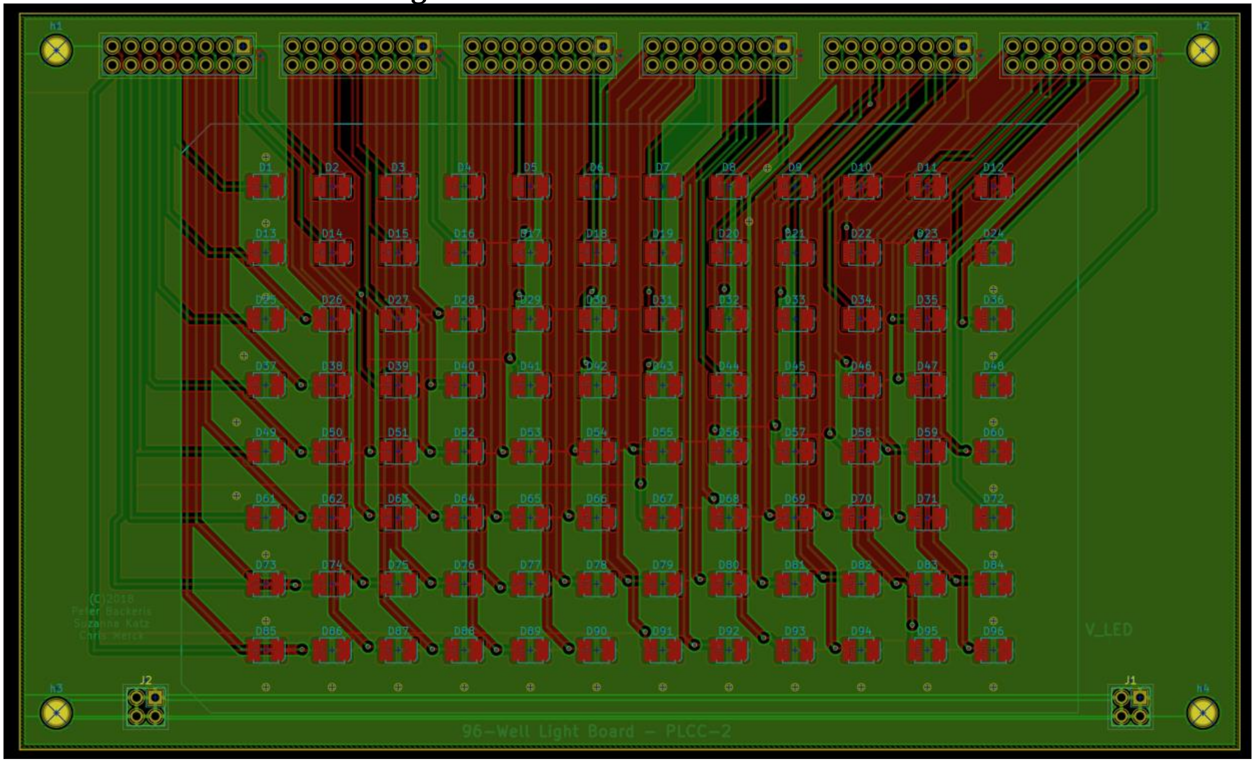

24-Well LED Board PCB designed in KiCAD

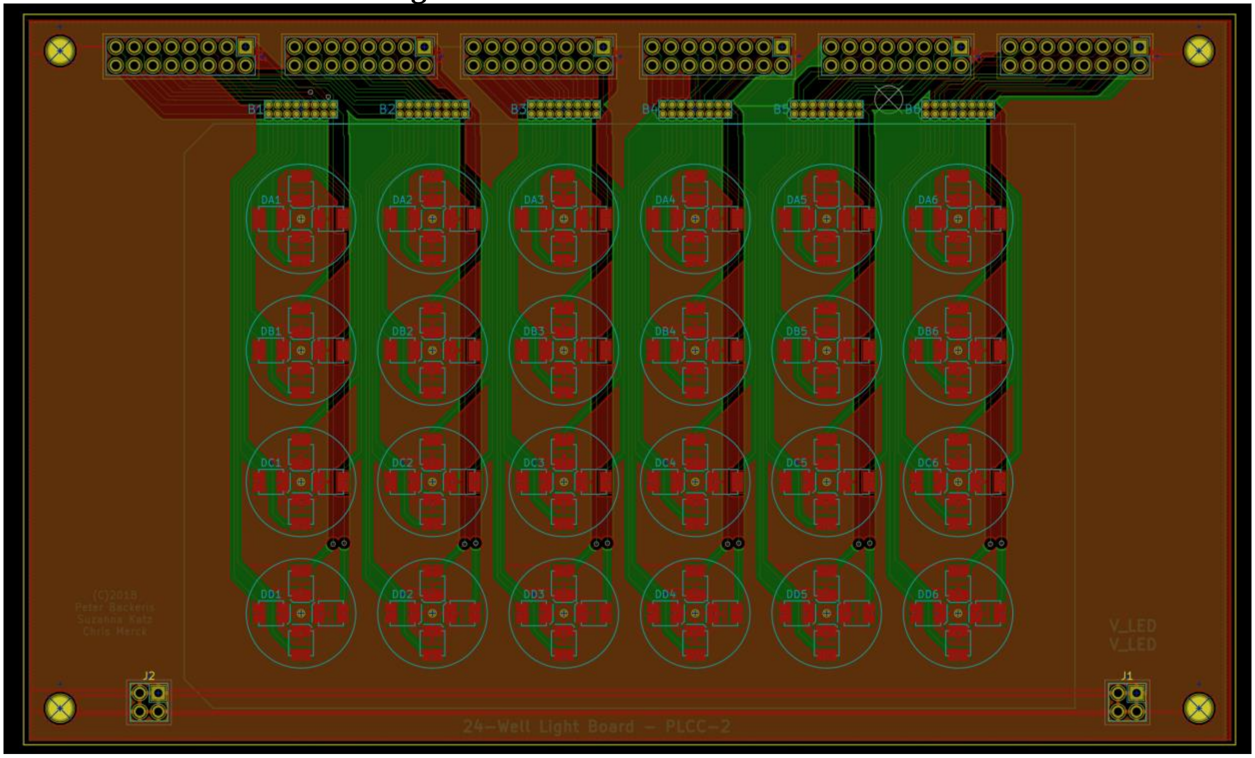

LED platform and MPS base - Design and Fabrication

The base of the MPS, as well as the isolation plate for the LED platform were designed in Solidworks and fabricated in-house from acrylic sheets. The base for the MPS is a 3/16” sheet of clear acrylic cut laser-cut exactly to the dimensions of the LED control board, with a hole in each corner aligned to the holes in the control board. The holes were tapped with #4-40 thread to allow 1” 4-40 aluminum hex standoffs to be fixed in each corner. The control board is aligned over the standoffs and screwed in place by another set of standoffs.

The isolation plate is constructed from 2 laser-cut sheets of black acrylic. The first layer (3/16” thick) is cut to fit over the LED board, including holes for each LED well, and through-holes in the corners for screws to sandwich the isolation plate to the standoffs above the LED control board. The second layer (1/8” thick) is cut to fit to the bottom of a particular 96 or 24-well microplate. These can be interchanged and redesigned accordingly to accommodate virtually any commercially available microplate. The two-layers are aligned using the led well holes and then bonded together using methylene chloride-based plastic welder. The isolation plate is stacked on top of the LED board to form the LED platform which is screwed down to the standoffs on the MPS.

Bill of Materials for 96-well or 24-well, 405nm MPS

Line #	Description	Manufacturer	Manufacturer Part Number	Supplier	Supllier Catalog Number	Unit Price	Qty	Subtotal
1	LED Control Board PCB	GoldPhoenix PCB	custom	GoldPhoenix PCB	--	$15.00	1	$15.00
2	96 or 24-well LED board	GoldPhoenix PCB	custom	GoldPhoenix PCB	--	$15.00	1	$15.00
3	TLC59401 Breakout Board	GoldPhoenix PCB	custom	GoldPhoenix PCB	--	$2.00	6	$12.00
4	ATX Breakout Board	Cytron	--	eBay	--	$10.00	1	$10.00
5	Teensy 2.0	PJRC	Teensy 2.0	PJRC	TEENSY	$19.00	1	$19.00
6	Adafruit Huzzah ESP8266 Breakout	Adafruit	2471	Adafruit	2471	$9.95	1	$9.95
7	Standard PLCC-2 405nm UV LED	Vishay	VLMU3100-GS08	Mouser	78-VLMU3100-GS08	$0.32	96	$30.43
8	TERM BLOCK HDR 3POS 90DEG 5MM	On Shore Technology	OSTOQ037151	Digi-Key	ED2792-ND	$0.39	1	$0.39
9	TERM BLOCK PLUG 3POS STR 5MM	On Shore Technology	OSTTJ037150	Digi-Key	ED2780-ND	$1.22	1	$1.22
10	RECEPT .100" DUAL ROW 16 POS	On Shore Technology	SH2-16G	Digi-Key	ED3071-ND	$0.35	12	$4.14
11	CONN HEADER FMAL 4PS .1" DL GOLD	Sullins Connector Solutions	PPPC022LFBN-RC	Digi-Key	S7105-ND	$0.57	4	$2.28
12	2x40 Header .1sp .235 Tails both sides	--	--	PHOENIX ENTERPRISES	HWS15781	$2.06	2	$4.12
13	10' 5 Cond 18 Gauge 0.156" 3.96mm Spacing Flat Ribbon Cable 5C 18AWG	3M	8132/05	eBay	--	$7.95	1	$7.95
14	4-40 Standoffs 1/4" Hex 2.25" length	--	--	McMaster-Carr	93505A004	$0.98	4	$3.92
15	4-40 Standoffs 1/4" Hex .75" length	--	--	McMaster-Carr	93505A434	$0.51	4	$2.04
16	Acrylic (All Components)	*fabricated	*fabricated	McMaster-Carr	--	$15.00	1	$15.00
17	Misc. components - resistors, capacitors, jumpers, solder, leds, screws, magnets, etc	--	--	--	--	$15.00	1	$15.00
18	Corsair CX430 80W ATX PSU	Corsair	CP-9020047-US	Amazon	--	$45.00	1	$45.00
			**MPS Total Cost of Materials:**					$212.44

The cost of 1 MPS system is approximately $212.44. If the power supply can be shared, the cost of the second system would be approximately $158.27.
