# Supplementary material for: Design and validation of an open-source modular Microplate Photoirradiation System for high-throughput photobiology experiments
Source: PLoS One. 2018 Oct 5;13(10):e0203597. doi: 10.1371/journal.pone.0203597 (PMC6173374; doi:10.1371/journal.pone.0203597)

S2. LED CONTROL AND CALIBRATION

Description of LED PWM and Current Control of Irradiance

The TLC59401 LED drivers have two modes of controlling LED intensity: pulse-width modulation (PWM) and current. PWM is method for controlling the duty-cycle of a digital pulse signal by modulating the duration (pulse width) of the high-state of the signal, which cycles on and off at a fixed, high frequency (1 kHz in the current configuration). The current level controls instantaneous amplitude of the digital pulse (instantaneous current to the LED). Figure S2.1 shows how these two parameters modulate the signal to the LED, which together control the average power provided to the LED in a precise manner. The TLC59401 has 6-bit (64 levels) of current control and 12-bit (4096 levels) of PWM duty cycle control which can be set at high speed from the Teensy 2.0 microcontroller board.

Figure S2.1 LED irradiance intensity control through modulation of pulse width (t_n) and current amplitude (I_n)

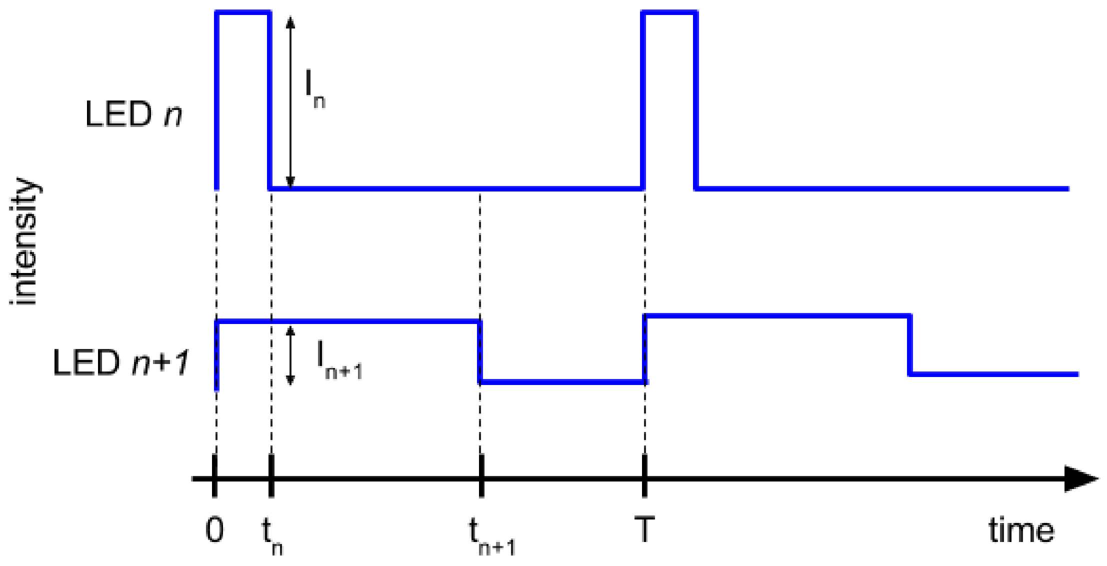

LED Calibration System

A calibration software utility for Windows was developed in Visual Studio 2017 (.Net C# WPF), to semi-automate the calibration process using a ThorLabs PM100USB Power Meter Interface connected with ThorLabs S121C Photodiode Sensor (400-1100 nm range). A custom laser-cut alignment disc was fabricated from black acrylic to center the sensor over each isolation plate opening (See Figure S2.2).

Figure S2.2 Photograph of the calibration system components

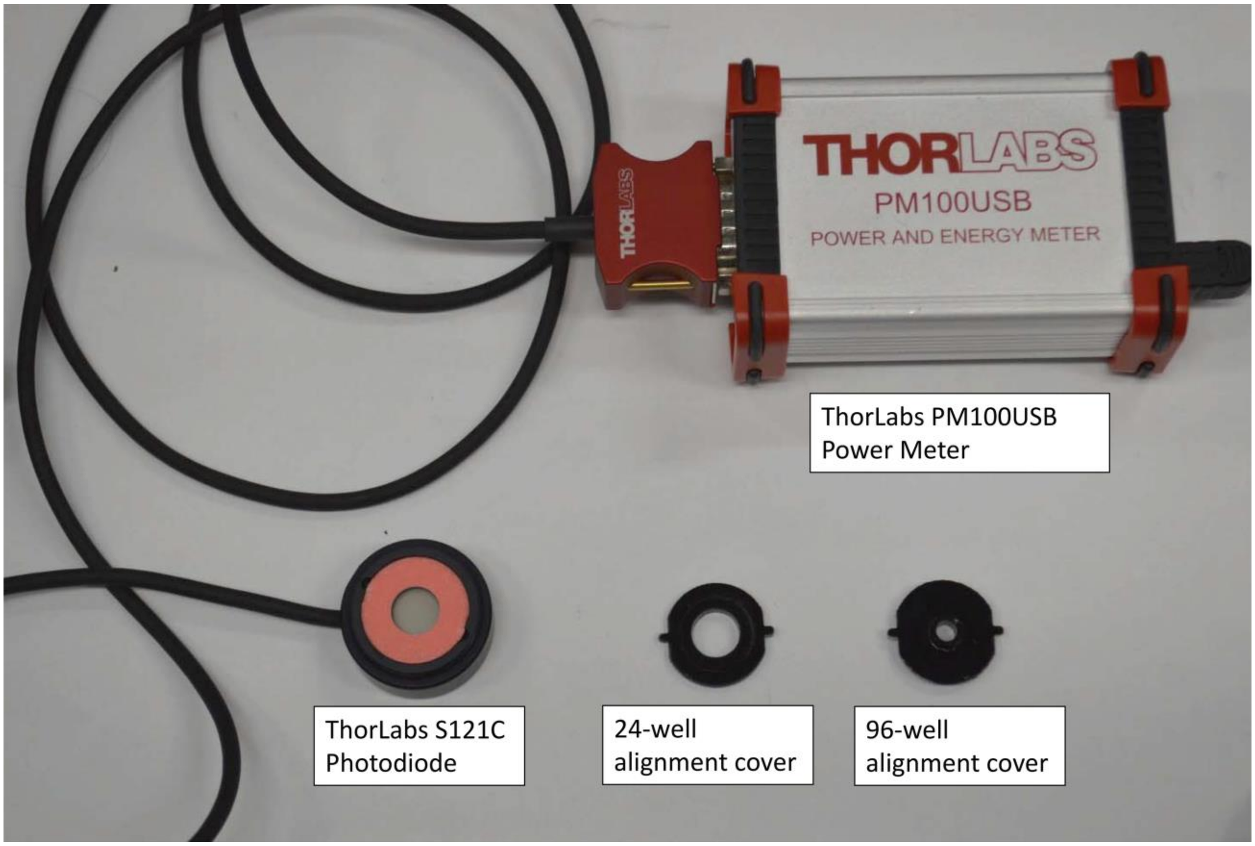

The alignment discs feature a protruding hollow cylinder in the center of the disc that fits snugly into the openings of the isolation plate to position the sensor over the center of the LED, at a height equivalent to the height of the bottom of a microplate well. The software features a GUI that interfaces with both the Teensy 2.0 (through USB Serial Port connection) and the PM100USB. The user has options to configure 24 vs 96-well plate format, and to set the wavelength of the LEDs being measured. The user then selects a channel number to calibrate and places the S121C sensor with alignment disc over the corresponding channel on the isolation plate. When ready, the user presses the “calibrate” button in the software to initiate a process by which the channel’s dot-correction is stepped through the values 0 up to the max of 63, while holding the PWM at max (4095). At each step, the value is held for 300ms while the value from the PM100USB is read and stored in an array. The raw power intensity values are converted to mW/cm2 and plotted against dot-correction value in a graph, along with a quadratic line of best fit calculated using linear regression algorithm. The three coefficient values are automatically displayed in text fields in the calibration utility, which can then be sent to the Teensy 2.0 over the USB Serial Port connection and written to EEPROM (for long-term storage) by clicking the “Update” button. A quadratic equation was used instead of a linear model due to a slight curvature in the graph that caused increasing error towards lower and higher values in the range of the LED. A quadratic fit was found to reduce this error and resulted in accurate irradiances across the entire range.

Figure S2.3 Screenshot of the MPS Calibration Software Utility, showing calibration curve output after automatically measuring an LED's irradiance over range of 0 to 63 dot-correction values.

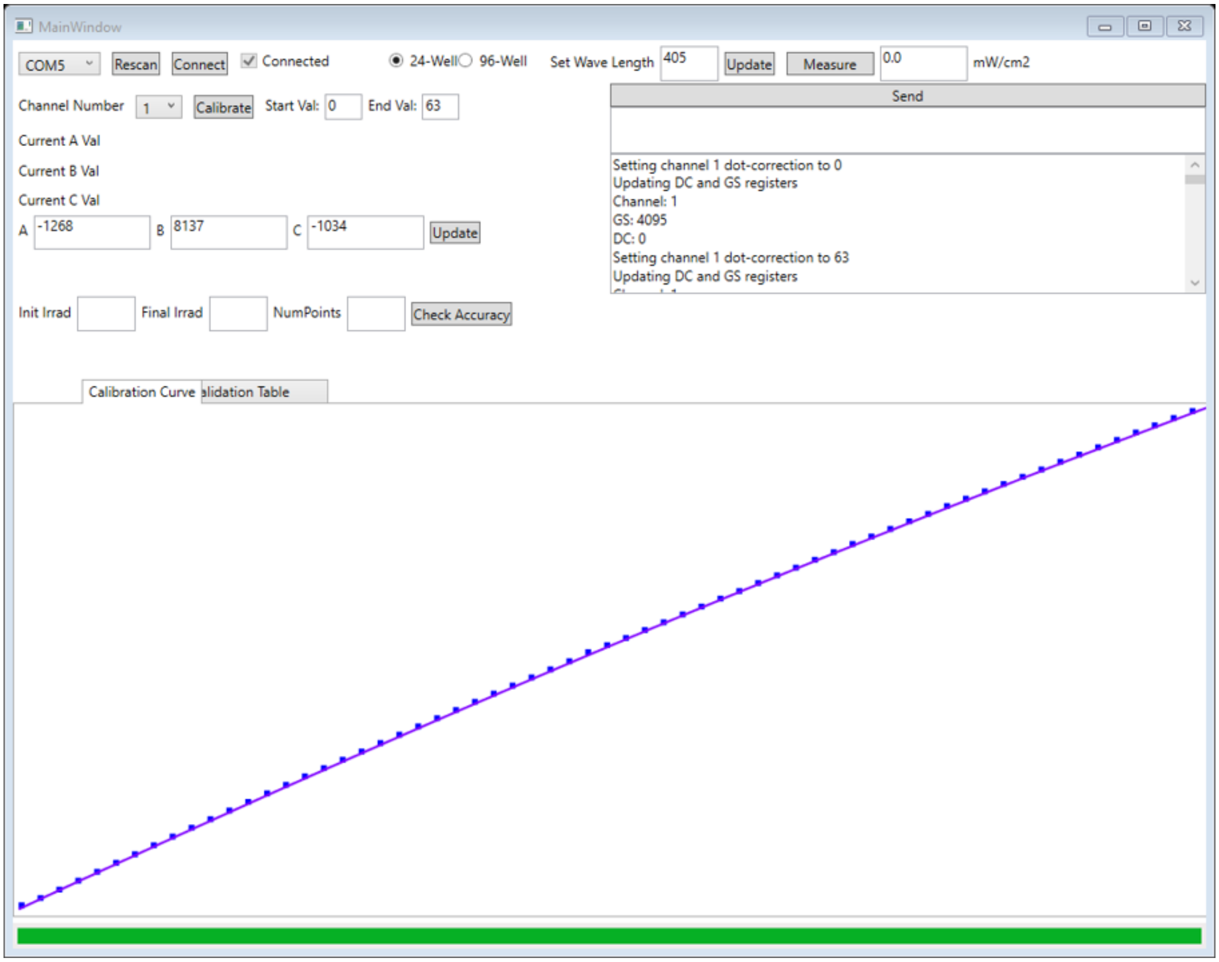

The Teensy 2.0 uses these 2nd order polynomial coefficients to calculate the current and PWM values to achieve the specified irradiance for each channel according to the following procedure:

1. First, the decimal current value is calculated using the formula:

   $$C_d = \frac{-B + \sqrt{B^2 - 4A(C - I_p)}}{2A}$$

   where C_d is the current value, I_p is the prescribed irradiance, A is the 2nd order coefficient, B is the first-order coefficient, and C is the zero-order coefficient .
2. C_d is rounded up to the nearest integer C_r, as the current value can only be set to integers between 0 and 63. If the value is above 63, an error is thrown to let the user know the LED is set to a value beyond its maximum possible.
3. The irradiance I_a at this rounded dot-correction value is calculated using the equation:

   $$I_a = A{C_r}^2 + BC_r + C$$
4. The PWM value is adjusted down from 4095 to compensate for this over-compensation of the current calibration using the formula: $P = \frac{I_p}{I_a} \times 4095$, where P is the PWM value, which is rounded to the nearest integer.

This procedure effectively matches current amplitude as closely as possible to coarsely reach the desired irradiance for each LED, then adjusts the PWM value from its maximum to finely match the prescribed irradiance as close as possible. The calibration utility also features the ability to check the accuracy of the calibration for each LED over a range of irradiance values that the user specifies through entering initial irradiance, final irradiance, and number of measurement points, which are used to determine the array of evenly spaced measurement

points. After setting these values and placing the sensor over the active LED, the user presses the Check Accuracy button which starts the process of sequentially setting the irradiance value for the LED and measuring the actual irradiance. The software records the measurements for each measurement point and displays the values for prescribed and measured irradiance in a table that lets the user copy and paste into excel for statistical analysis (See Figure S2.4).

Figure S2.4 Screenshot of the MPS Calibration Software Utility, showing validation data for 8 irradiance values between 0.5 and 15.5 mW/cm2. Table with prescribed irradiance and measured irradiance is provided for transferring to other analysis programs.

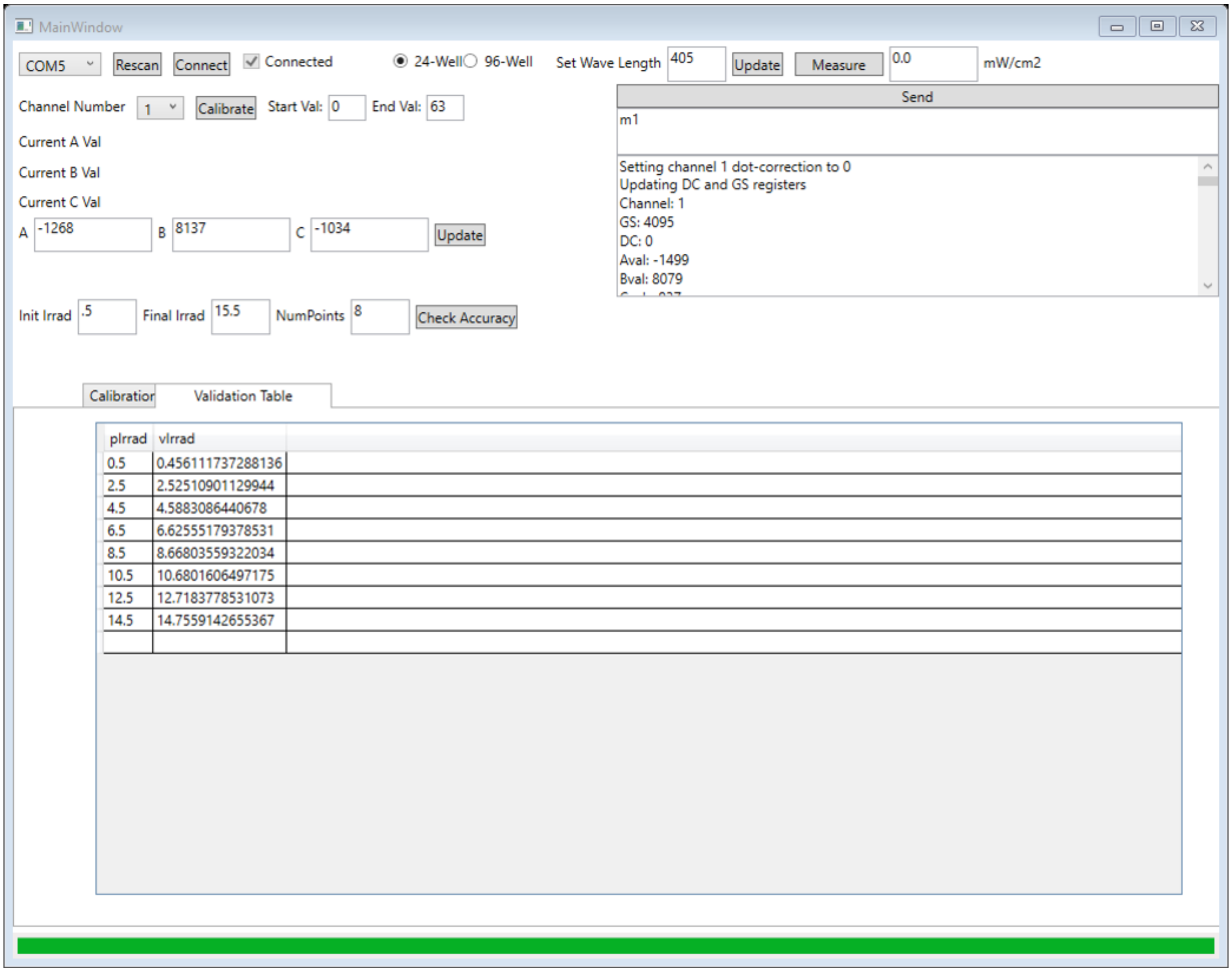

Using this method on both the 24-well and 96-well, 405-nm LED MPS's, calibration accuracy and precision were assessed and results are shown below in Table S2.1 and Table S2.2.

Table S2.1 Table of average validation data for 24-well 405nm MPS. Average measured irradiance of all LEDs for each of 8 prescribed irradiance values was calculated, along with other statistical measures of precision and accuracy.

24-well (immediately after calibration)					
Prescribed Irradiance (mW/cm^2)	Mean of Measured (mW/cm^2)	Std. Dev (mW/cm^2)	Coefficient of Variation (%)	Absolute Error (mW/cm^2)	Relative Error (%)
0.5	0.490352	0.417762	9.937199	0.068765	13.75296
2.5	2.452271	2.431826	0.405537	0.060264	2.410579
4.5	4.499662	4.434308	0.461556	0.045779	1.017316
6.5	6.537896	6.449828	0.446857	0.035334	0.543603
8.5	8.569092	8.443425	0.436032	0.02941	0.345995
10.5	10.58964	10.44324	0.427461	0.033832	0.322207
12.5	12.60866	12.43821	0.426639	0.040442	0.323535
14.5	14.62653	14.4277	0.414572	0.044155	0.304518

Table S2.2 Table of average validation data for 96-well 405nm MPS. Average measured irradiance of all LEDs for each of 8 prescribed irradiance values was calculated, along with other statistical measures of precision and accuracy. Validation measurements were repeated after a 3h stress test with all LEDs on to assess reliability of calibration after periods of use.

96-well (immediately after calibration)					
Prescribed Irradiance (mW/cm^2)	Mean of Measured (mW/cm^2)	Std. Dev (mW/cm^2)	Coefficient of Variation (%)	Absolute Error (mW/cm^2)	Relative Error (%)
0.5	0.468641	0.018093	3.860796	0.031511	6.302245
1.5	1.473736	0.01685	1.143327	0.026843	1.789509
2.5	2.477297	0.025633	1.034734	0.027637	1.105495
3.5	3.485359	0.034067	0.977418	0.028143	0.804098
4.5	4.492398	0.043223	0.962132	0.032437	0.720827
5.5	5.490448	0.052947	0.964353	0.039238	0.713415
6.5	6.489188	0.063021	0.971167	0.046634	0.717447
7.5	7.485787	0.072897	0.9738	0.054998	0.733302
96-well (after 3 hour stress test)					
Prescribed Irradiance (mW/cm^2)	Mean of Measured (mW/cm^2)	Std. Dev (mW/cm^2)	Coefficient of Variation (%)	Absolute Error (mW/cm^2)	Relative Error (%)
0.5	0.4582115	0.017933	3.913802	0.041788	8.357699
1.5	1.44319368	0.017249	1.195162	0.056806	3.787088
2.5	2.42789451	0.026098	1.074925	0.073001	2.920056
3.5	3.41651582	0.035227	1.031074	0.085077	2.430761
4.5	4.40456196	0.045337	1.029317	0.097748	2.172181
5.5	5.38374382	0.05601	1.040359	0.118979	2.163255
6.5	6.36378765	0.066261	1.041226	0.139279	2.142761
7.5	7.34111444	0.07598	1.034992	0.16248	2.166399

These results of the revalidation after stress test with the 96-well MPS reveal that there is some loss of accuracy, but not precision after periods of use. This could be caused by drift in LED output overtime, which may be possible to be reduced through a longer “burn-in” period for the LEDs which could stabilize the LED performance. The results also show a significantly greater error at the lower range of irradiance values for both 24-well and 96-well systems. This can

immediately be resolved by allocating certain wells in each plate to a specific range of irradiances (i.e. low, medium, high) and setting the calibration utility for those wells to have the corresponding start and end values of current for the calibration curve fitting.

Irradiance Radial Profile Analysis

The radial profile of light in individual wells in the MPS was analyzed as a measure of homogeneity of the light output on cells. A top-down image of each MPS with all wells set to the same irradiance, and with a sheet of paper covering the LED isolation plate was taken and loaded into ImageJ image analysis software (See Figure S2.5). The Radial Profile plugin was used to measure the average pixel intensity as a function of radius for each well, and the set of radial profile measurements for each well were normalized to their maximum values to give a relative intensity across each well. The measurements were averaged together for all wells and plotted along with standard deviations in Figures S2.6. Overall relative drop in irradiance from inner 10 percent of well radius to outer 10 percent of well radius was found to be 10% for the 96-well MPS, and less than 14 percent for the 24-well MPS.

Figure S2.5 Top-down image of 96-well and 24-well 405nm MPS's with all wells set to same irradiance (with sheet of paper over LED isolation plate) and used for irradiance radial profile measurement

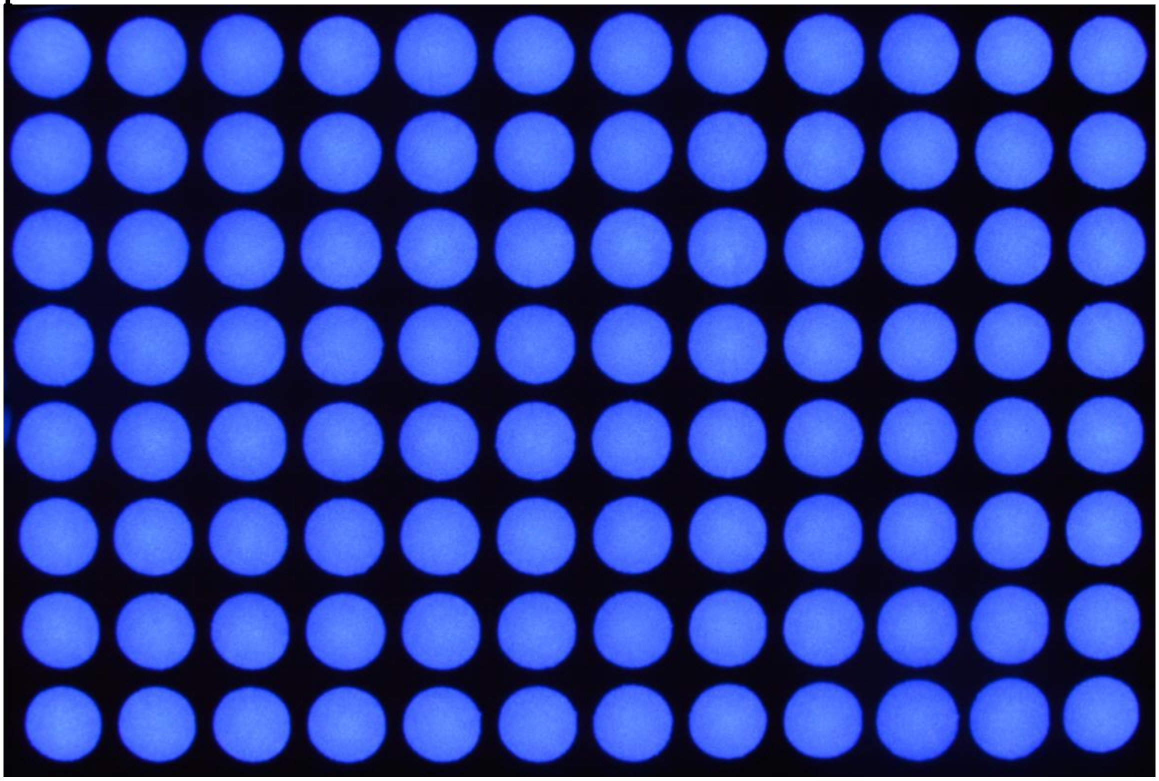

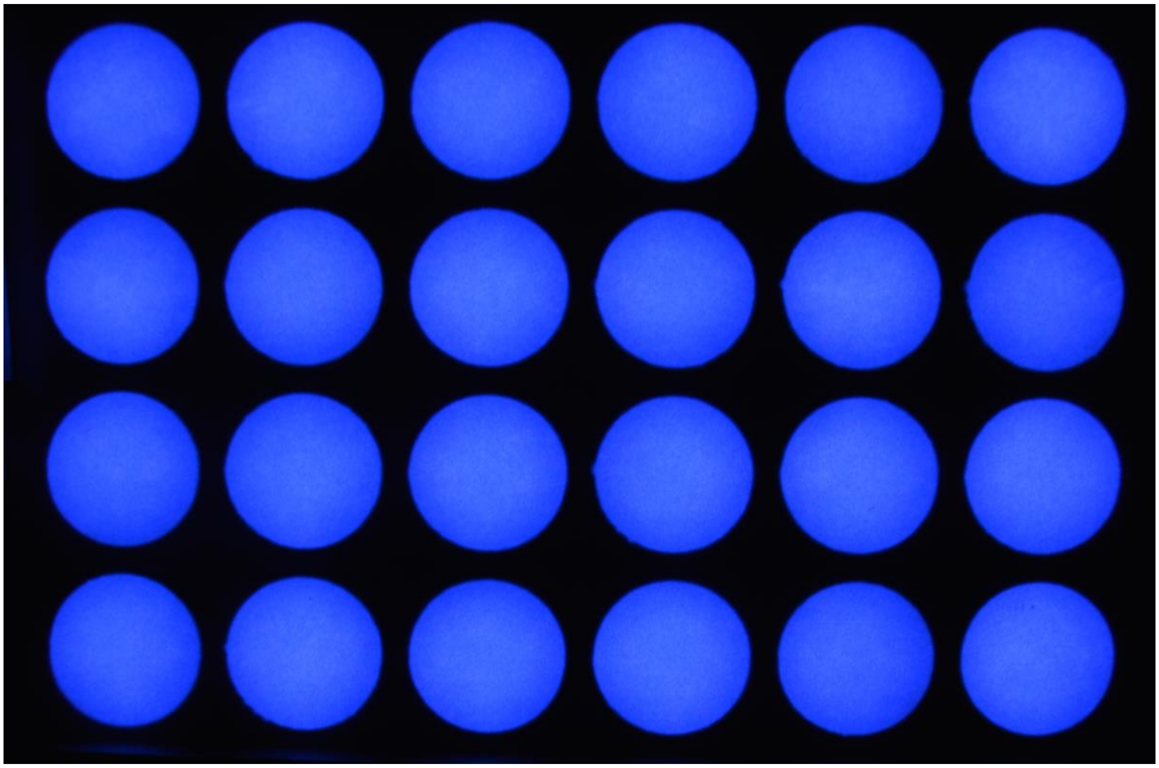

Figure S2.6 Graphs of average relative radial intensity across all wells in 96 and 24-well MPS, using photos in Figure S2.5

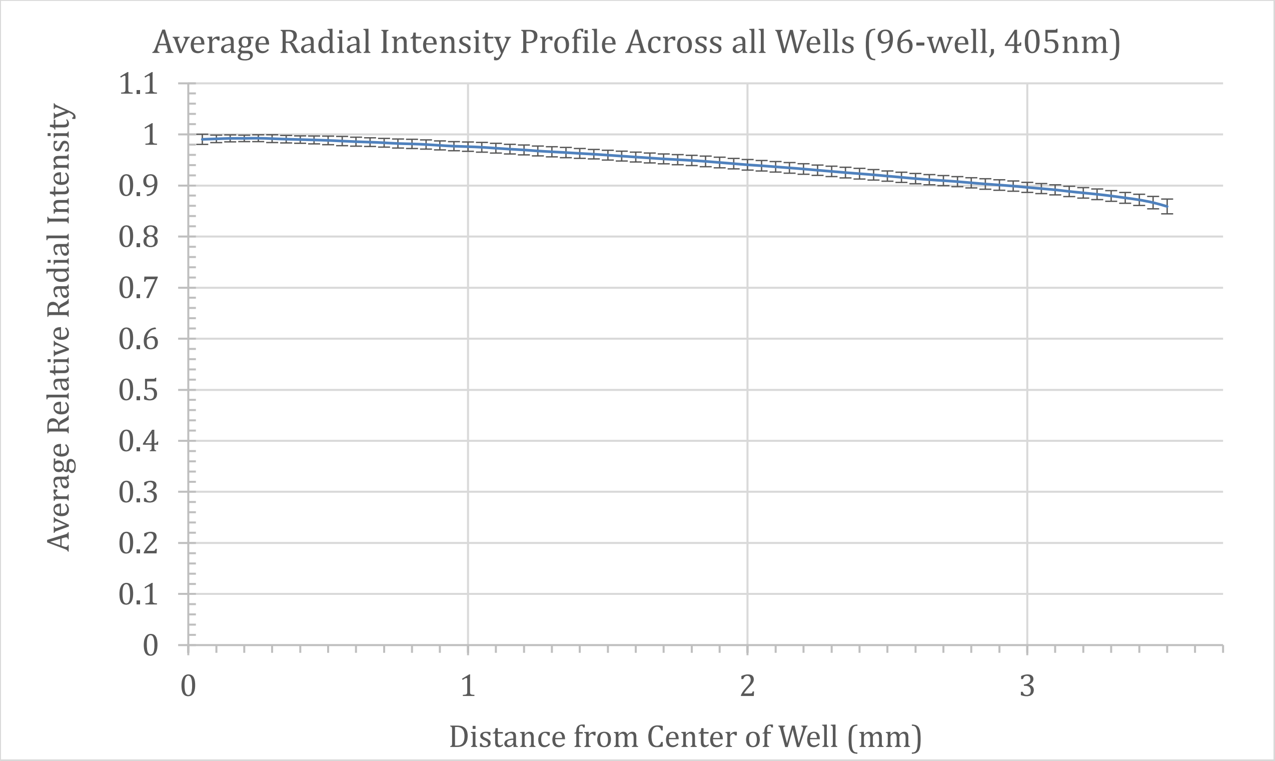

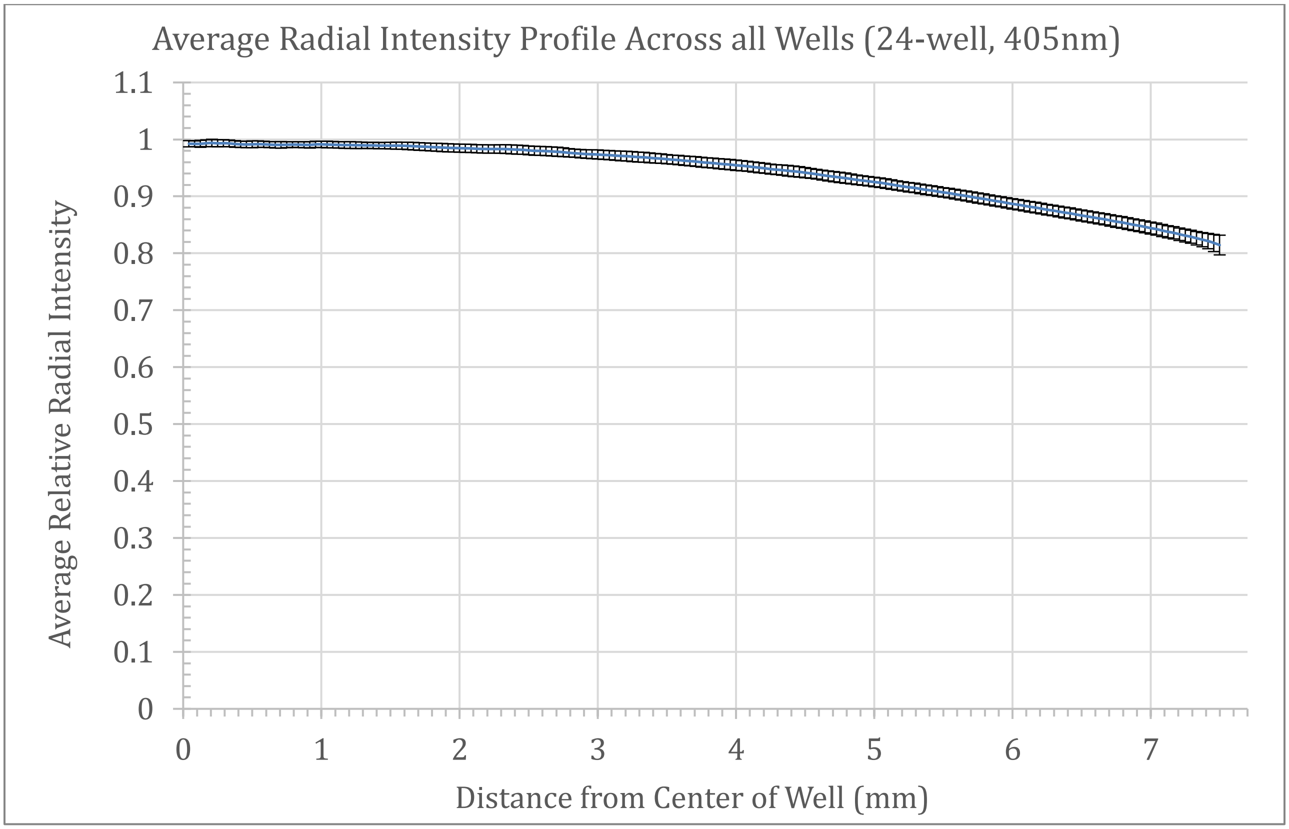

Supplement: S2 File — (PDF) [file pone.0203597.s002.pdf]
