# Supplementary material for: Design and validation of an open-source modular Microplate Photoirradiation System for high-throughput photobiology experiments
Source: PLoS One. 2018 Oct 5;13(10):e0203597. doi: 10.1371/journal.pone.0203597 (PMC6173374; doi:10.1371/journal.pone.0203597)
Supplement: S1 Table — (PDF) [file pone.0203597.s003.pdf]

S3. Light System Comparison Table

Comparison of our MPS with recent open-source microplate-based systems (green), as well as the commercially available LUMUS system (purple). Novel features contained in the MPS in comparison to other open-source designs are highlighted in blue.

Specification	MPS	Light Plate Apparatus	LED Illuminator	COMET	AXION LUMUS (commercial)
**Publication**	N/A	[1] Gerhardt et al. 2016	[2] Hannanta-anan & Chow. 20162	[3] Reis et al. 20163	[4] Clements et al. 2015
**Design Features**					
Format	both 24-well and 96-well	24-well	24-well	96-well	24 and 48-well culture plate (proprietary)
Throughput (Maximum Indpendent Light Conditions Per Plate)	24/96	24	6	8	24/48
LED Type	surface mount	through-hole	through-hole	through-hole	surface mount
LEDs per well	24-well=4; 96-well=1	2	2	1	4
Customizable LED range	customizable	customizable	customizable	customizable	fixed: (475, 530, 612, 655 nm)
**Control Features**					
Controller	Teensy 2.0 (Atmega32U4)	ATMega328a	Arduino (Model Unknown)	Arduino Mega	dual-core CPU with FPGA co-prossesor
LED Driver	TLC59401	TLC5940	PWM + potentiometer	CL25N3-G (fixed current 25 mA)	Unknown
Intensity Control	12-bit PWM, 6-bit Current Control	12-bit PWM, 6-bit Current Control	Analog/PWM	PWM	digital (driver unknown)
Waveform Amplitude Modulation	NO	YES	NO	NO	YES
Individual Well Control	YES	YES	NO	NO	YES
Firmware Source Code Documentation	open-source	open-source	open-source	open-source	closed-source
Graphical User Inteface	Yes; web browser-based	Yes; web broswer-based (Iris)	No; Arduino IDE (Serial Interface)	No; Arduino IDE (Serial Interface)	Yes; PC software (AxIS)
Program Transfer Method/Storage	Wireless Web App (USB serial optional)	SD card	None reported	None (Firmware must be re-reprogrammed for each experiment)	USB to device memory
Environmental Control	Yes; can be placed inside incubator	Yes; can be placed inside incubator	Yes; can be placed inside incubator	Yes; can be placed inside incubator	Yes; system equipped with its own controlled environment
Remote/Wireless control	YES; WiFi	NO	NO	NO	NO
**Hardware Fabrication**					
Stage/Platform setup	Modular Laser-cut acrylic stage and replaceable isolation plate with aluminum stand-offs	3D-printed ABS	Laser-cut acrylic	laser-cut acrylic with water-jet cut copper block as a heat-sink	N/A
Assembly Cost	$200-250	$150-400	not reported	not reported	$26,000
Hardware Design and Fabrication Documentation	Open	Open	Open	Open	Proprietary
**Calibration**					
LED Calibration	YES	YES	Limited	not reported	unknown
Calibration Memory	YES; EEPROM	SD card	NO	NO	unknown
Automated Measurement	Custom GUI for automatically measuring irradiance and fitting to driver output for each LED channel	MATLAB image analysis to calibrate relative brightness to known irradiance. Manual calibration with spectrometer of 5 LEDs to calibrate image brightness to true values	Manual - Analog potentiometer adjustement and LED quality control	N/A	unknown
Device-level Irradiance Control	Yes - Custom GUI outputs quadratic calibration parameters for each LED to device for automated calculation of settings for any desired irradiance	No- Single-value calibration of each LED for one experiment - must repeat calibration procedure for new irradiance settings	N/A	N/A	unknown
Accessory Instruments	ThorLabs S121C Photodiode Power Sensor (400-1100 nm) connected to a Thorlabs PM100USB Power Meter Interface with custom laser-cut well adaptors (24 and 96-well)	Pixel analysis in with a camera in an enclosed gel-imager (FluorChem FC2, Alpha Innotech) + probe spectrometer (StellarNet Inc, photodetector: EPP2000 UVN-SR-25LT-16, probe: F600-UV-VIS-SR, software: SpectraWiz)	ThorLabs PM100D power meter with S120 sensor	NO	uknown
Calibration Documentation	Yes	Yes	NO	NO	unknown

Specification	MPS	Light Plate Apparatus	LED Illuminator	COMET	AXION LUMUS (commercial)
Publication	N/A	[1] Gerhardt et al. 2016	[2] Hannanta-anan & Chow. 20162	[3] Reis et al. 20163	[4] Clements et al. 2015
Design Features					
Format	both 24-well and 96-well	24-well	24-well	96-well	24 and 48-well culture plate (proprietary)
Throughput (Maximum Indpendent Light Conditions Per Plate)	24/96	24	6	8	24/48
LED Type	surface mount	through-hole	through-hole	through-hole	surface mount
LEDs per well	24-well=4; 96-well=1	2	2	1	4
Customizable LED range	customizable	customizable	customizable	customizable	fixed: (475, 530, 612, 655 nm)
Control Features					
Controller	Teensy 2.0 (Atmega32U4)	ATMega328a	Arduino (Model Unknown)	Arduino Mega	dual-core CPU with FPGA co-prossesor
LED Driver	TLC59401	TLC5940	PWM + potentiometer	CL25N3-G (fixed current 25 mA)	Unknown
Intensity Control	12-bit PWM, 6-bit Current Control	12-bit PWM, 6-bit Current Control	Analog/PWM	PWM	digital (driver unknown)
Waveform Amplitude Modulation	NO	YES	NO	NO	YES
Individual Well Control	YES	YES	NO	NO	YES
Firmware Source Code Documentation	open-source	open-source	open-source	open-source	closed-source
Graphical User Inteface	Yes; web browser-based	Yes; web broswer-based (Iris)	No; Arduino IDE (Serial Interface)	No; Arduino IDE (Serial Interface)	Yes; PC software (AxIS)
Program Transfer Method/Storage	Wireless Web App (USB serial optional)	SD card	None reported	None (Firmware must be re-reprogrammed for each experiment)	USB to device memory
Environmental Control	Yes; can be placed inside incubator	Yes; can be placed inside incubator	Yes; can be placed inside incubator	Yes; can be placed inside incubator	Yes; system equipped with its own controlled environment
Remote/Wireless control	YES; WiFi	NO	NO	NO	NO
Hardware Fabrication					
Stage/Platform setup	Modular Laser-cut acrylic stage and replaceable isolation plate with aluminum stand-offs	3D-printed ABS	Laser-cut acrylic	laser-cut acrylic with water-jet cut copper block as a heat-sink	N/A
Assembly Cost	$200-250	$150-400	not reported	not reported	$26,000
Hardware Design and Fabrication Documentation	Open	Open	Open	Open	Proprietary
Calibration					
LED Calibration	YES	YES	Limited	not reported	unknown
Calibration Memory	YES; EEPROM	SD card	NO	NO	unknown
Automated Measurement	Custom GUI for automatically measuring irradiance and fitting to driver output for each LED channel	MATLAB image analysis to calibrate relative brightness to known irradiance. Manual calibration with spectrometer of 5 LEDs to calibrate image brightness to true values	Manual - Analog potentiometer adjustement and LED quality control	N/A	unknown
Device-level Irradiance Control	Yes - Custom GUI outputs quadratic calibration parameters for each LED to device for automated calculation of settings for any desired irradiance	No- Single-value calibration of each LED for one experiment - must repeat calibration procedure for new irradiance settings	N/A	N/A	unknown
Accessory Instruments	ThorLabs S121C Photodiode Power Sensor (400-1100 nm) connected to a Thorlabs PM100USB Power Meter Interface with custom laser-cut well adaptors (24 and 96-well)	Pixel analysis in with a camera in an enclosed gel-imager (FluorChem FC2, Alpha Innotech) + probe spectrometer (StellarNet Inc, photodetector: EPP2000 UVN-SR-25LT-16, probe: F600-UV-VIS-SR, software: SpectraWiz)	ThorLabs PM100D power meter with S120 sensor	NO	uknown
Calibration Documentation	Yes	Yes	NO	NO	unknown

References

1. Gerhardt, K. P., Olson, E. J., Castillo-Hair, S. M., Hartsough, L. A., Landry, B. P., Ekness, F., et al. An open-hardware platform for optogenetics and photobiology. Scientific Reports .

2. Hannanta-anan P, Chow B. Optogenetic Control of Calcium Oscillation Waveform Defines NFAT as an Integrator of Calcium Load. Cell Systems. 2016;2(4):283–8.

3. Reis, S. A., Ghosh, B., Hendricks, A. J., Szantai-Kis, M. D., Törk, L., Ross, K. N., et al. Light-controlled modulation of gene expression by chemical optoepigenetic probes. Nature Chemical Biology. 2016, 12 (5), 317-323.

4. Clements, I.P, et al. Optogenetic stimulation of multiwell MEA plates for neural and cardiac applications. Proc. of SPIE, 2016, Vol. 9690 96902C-1.
