# Supplementary material for: Design and validation of an open-source modular Microplate Photoirradiation System for high-throughput photobiology experiments
Source: PLoS One. 2018 Oct 5;13(10):e0203597. doi: 10.1371/journal.pone.0203597 (PMC6173374; doi:10.1371/journal.pone.0203597)
Supplement: S3 File — (PDF) [file pone.0203597.s004.pdf]

S4. Experimental Parameters and Plate Configurations for Figure 5 and 6 graphs.

“Increasing Duration/Pulsation“ Configuration

	1	2	3	4	5	6	7	8	9	10	11	12
A	ALA, no light						No ALA, no Light					
B		ALA, 1.25min I=1.5mW/cm$_2$ (Fluence = .1J/cm$_2$)							ALA, 2.5min, I=1.5mW/cm$_2$ (Fluence = .2J/cm$_2$)			
C			ALA, 5min, I=1.5mW/cm$_2$ (Fluence = .5J/cm$_2$)									
D	ALA, 10min, I=1.5mW/cm$_2$ (Fluence = .9J/cm$_2$)					ALA, 20min, I=1.5mW/cm$_2$ (Fluence = 1.8J/cm$_2$)						
E	ALA, Duty Cycle=25%, 25Hz, I=4.5mW/cm$_2$, Fluence = 1.35J/cm$_2$						ALA, Duty Cycle=50%, 10Hz, I=4.5mW/cm$_2$, Fluence = 1.35J/cm$_2$					
F	ALA, Duty Cycle=25%, 5Hz, I=4.5mW/cm$_2$, Fluence = 1.35J/cm$_2$						ALA, Duty Cycle=50%, 5Hz, I=4.5mW/cm$_2$, Fluence = 1.35J/cm$_2$					
G	ALA, Duty Cycle=33.3%, 3.33Hz, I=4.5mW/cm$_2$, Fluence = 1.35J/cm$_2$						ALA, Duty Cycle=25%, 1Hz, I=4.5mW/cm$_2$, Fluence = 1.35J/cm$_2$					
H	ALA, Duty Cycle=25%, .5Hz, I=4.5mW/cm$_2$, Fluence = 1.35J/cm$_2$						ALA, Duty Cycle=100%, 5min, 1 cycle (0Hz), I=4.5mW/cm$_2$, Fluence = 1.35J/cm$_2$					

“Fluence” 24-well Configuration

	1	2	3	4	5	6	7	8	9	10	11	12
A	ALA, no light						No ALA, no Light					
B	1.25min, I=6mW/cm^2				ALA, 2.5min, I=.75mW/cm^2				ALA, 5min, I=.75mW/cm^2			
C	ALA, 10min, I=.75mW/cm^2				ALA, 20min, I=.75mW/cm^2				ALA, 2.5min, I=1.5mW/cm^2			
D	ALA, 5min, I=1.5mW/cm^2				ALA, 10min, I=1.5mW/cm^2				ALA, 20min, I=1.5mW/cm^2			
E	ALA, 2.5min, I=3mW/cm^2				ALA, 5min, I=3mW/cm^2				ALA, 10min, I=3mW/cm^2			
F	ALA, 20min, I=3mW/cm^2				ALA, 2.5min, I=4.5mW/cm^2				ALA, 5min, I=4.5mW/cm^2			
G	ALA, 10min, I=4.5mW/cm^2				ALA, 20min, I=4.5mW/cm^2				ALA, 2.5min, I=6mW/cm^2			
H	ALA, 5min, I=6mW/cm^2				ALA, 10min, I=6mW/cm^2				ALA, 20min, I=6mW/cm^2			

Duration	0	150	300	150	75	600	300	150	150	1200	600	300	150	300	1200	600	300	600	1200	600	1200	1200	Seconds
Irradiance	0	0.75	0.75	1.5	6	0.75	1.5	3	4.5	0.75	1.5	3	6	4.5	1.5	3	6	4.5	3	6	4.5	6	mW/cm^2
Fluence	0	0.1125	0.225	0.225	0.45	0.45	0.45	0.45	0.675	0.9	0.9	0.9	0.9	1.35	1.8	1.8	1.8	2.7	3.6	3.6	5.4	7.2	J/cm^2

S4. Experimental Parameters and Plate Configurations for Figure 5 and 6 graphs.

“Increasing Irradiance/Pulsation“ Configuration

<table>
<tr><th></th><th>1</th><th>2</th><th>3</th><th>4</th><th>5</th><th>6</th><th>7</th><th>8</th><th>9</th><th>10</th><th>11</th><th>12</th></tr>
<tr><th>A</th><td colspan="6">ALA, no light</td><td colspan="6">No ALA, no Light</td></tr>
<tr><th>B</th><td>(ALA, no light)</td><td colspan="7">ALA, 20min, I=.75mW/cm_2 (Fluence .9J/cm_2)</td><td colspan="4" rowspan="2">ALA, 20min, I=1.5mW/cm_2 (Fluence = 1.8J/cm_2)</td></tr>
<tr><th>C</th><td colspan="2" rowspan="2">ALA, 20min, I=4.5mW/cm_2 (Fluence = 5.4J/cm_2)</td><td colspan="7">ALA, 20min, I=3mW/cm_2 (Fluence = 3.6J/cm_2)</td></tr>
<tr><th>D</th><td colspan="3">(ALA, 20min, I=4.5mW/cm_2)</td><td colspan="7">ALA, 20min, I=6mW/cm_2 (Fluence = 7.2J/cm_2)</td></tr>
<tr><th>E</th><td colspan="6">ALA, Duty Cycle=25%, 25Hz, I=4.5mW/cm_2, Fluence = 1.35J/cm_2</td><td colspan="6">ALA, Duty Cycle=50%, 10Hz, I=4.5mW/cm_2, Fluence = 1.35J/cm_2</td></tr>
<tr><th>F</th><td colspan="6">ALA, Duty Cycle=25%, 5Hz, I=4.5mW/cm_2, Fluence = 1.35J/cm_2</td><td colspan="6">ALA, Duty Cycle=50%, 5Hz, I=4.5mW/cm_2, Fluence = 1.35J/cm_2</td></tr>
<tr><th>G</th><td colspan="6">ALA, Duty Cycle=33.3%, 3.33Hz, I=4.5mW/cm_2, Fluence = 1.35J/cm_2</td><td colspan="6">ALA, Duty Cycle=25%, 1Hz, I=4.5mW/cm_2, Fluence = 1.35J/cm_2</td></tr>
<tr><th>H</th><td colspan="6">ALA, Duty Cycle=25%, .5Hz, I=4.5mW/cm_2, Fluence = 1.35J/cm_2</td><td colspan="6">ALA, Duty Cycle=100%, 5min, 1 cycle (0Hz), I=4.5mW/cm_2, Fluence = 1.35J/cm_2</td></tr>
</table>

“Increasing Irradiance” 24-well Configuration

<table>
<tr><th></th><th>1</th><th>2</th><th>3</th><th>4</th><th>5</th><th>6</th></tr>
<tr><th>A</th><td colspan="6">No ALA, no Light</td></tr>
<tr><th>B</th><td colspan="3">ALA, no light</td><td colspan="3">ALA, 20min, I=.75mW/cm2 (Fluence .9J/cm2)</td></tr>
<tr><th>C</th><td colspan="3">ALA, 20min, I=1.5mW/cm2 (Fluence = 1.8J/cm2)</td><td colspan="3">ALA, 20min, I=3mW/cm2 (Fluence = 3.6J/cm2)</td></tr>
<tr><th>D</th><td colspan="3">ALA, 20min, I=4.5mW/cm2 (Fluence = 5.4J/cm2)</td><td colspan="3">ALA, 20min, I=6mW/cm2 (Fluence = 7.2J/cm2)</td></tr>
</table>

S4. Microplate Configurations/batches used in Figure 5 graphs.

Below are the two microplate configurations used in the **Figure 5C** graph in which 96-well (top) and 24-well (bottom) microplate formats are used to show PpIX photobleaching with increasing fluence (by increasing irradiance at a fixed duration of 1200s). Only the top half of the 96-well plate (rows A-D) were used for this graph (the bottom contains pulsation experiments used in a Figure5G). 96-well Experiments used in the comparison are: plate/batch id: 2438, 2439, 2477. The 24-well microplate format contains the same parameters as the 96-well, at 24h ALA incubation, increasing radiant exposure (Fluence shown in parantheses) by increasing irradiance at a fixed duration of 1200s. 24-well Experiments used in the **Figure 5C** comparison are: plate/batch id: 2442, 2444, 2445.

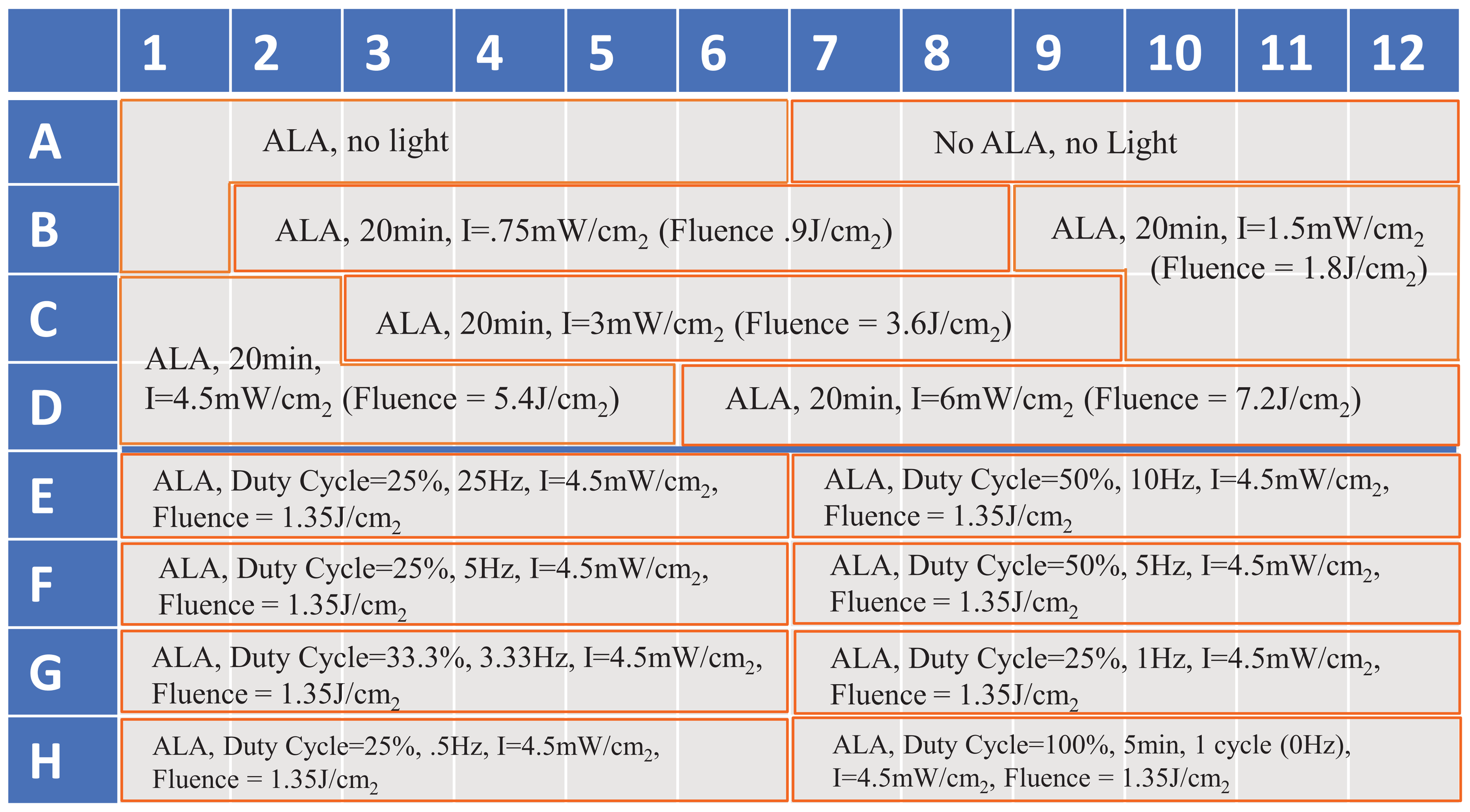

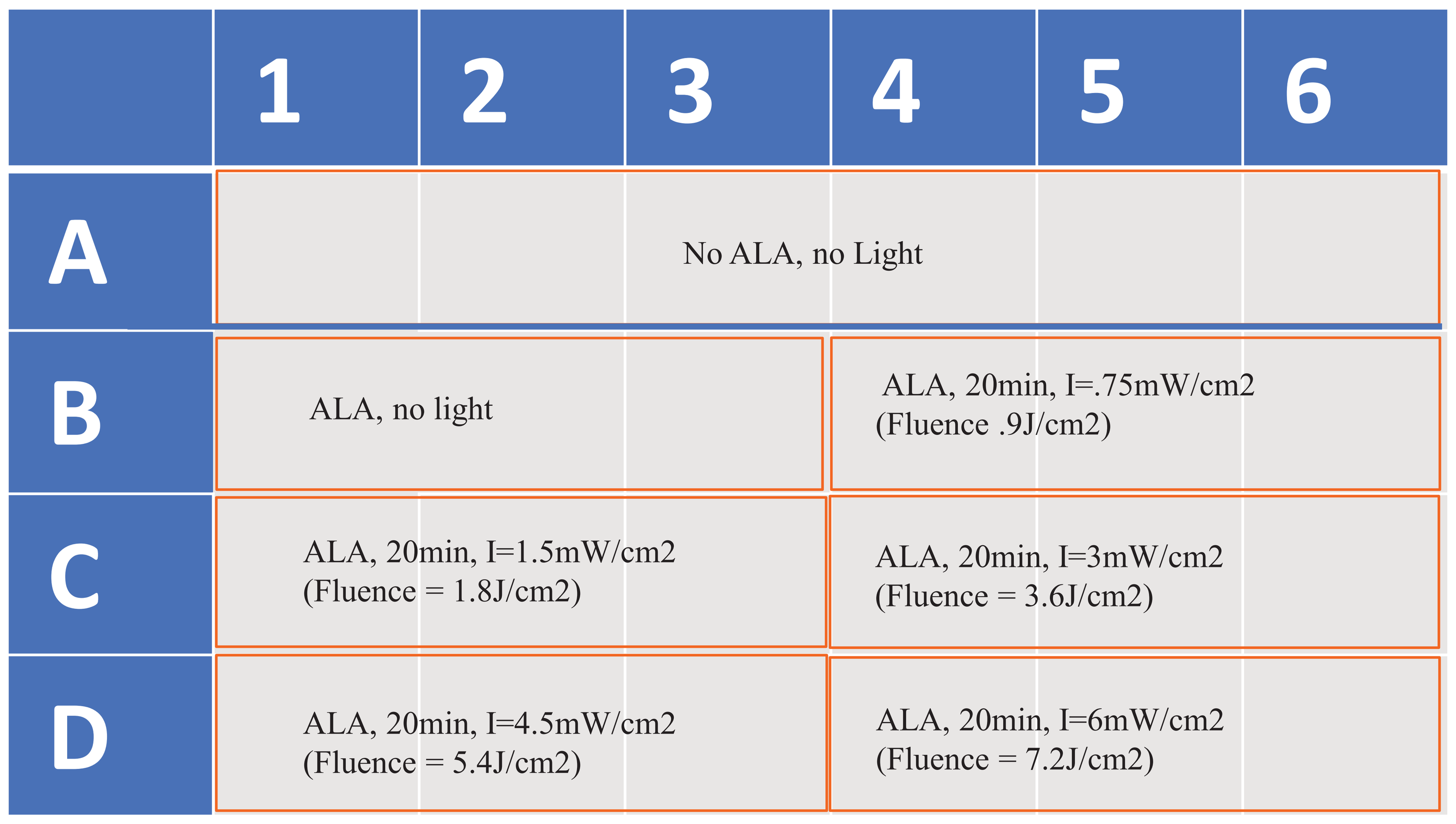

Below are the two microplate configurations used in the **Figure 5D, 5E, and 5F** graphs which are used to show PpIX photobleaching with increasing fluence (by increasing duration at a fixed irradiance of 1.5mW/cm2). Only the top half of the "Increasing Irradiance" 96-well plate configuration (rows A-D) were used for this graph, combined with the same conditions found in the 96-well "Fluence" configuration. Experiments used in the figure are: 6h ALA incubation graph D: **2399, 2466 2467** 2506 2508; 24h ALA incubation graph E: **2368, 2435, 2476**, 2485, 2486; 36h ALA incubation graph F: 2492, 2493, 2497, **2480, 2481, 2382.** The batches using the "Fluence" configuration are in bold.

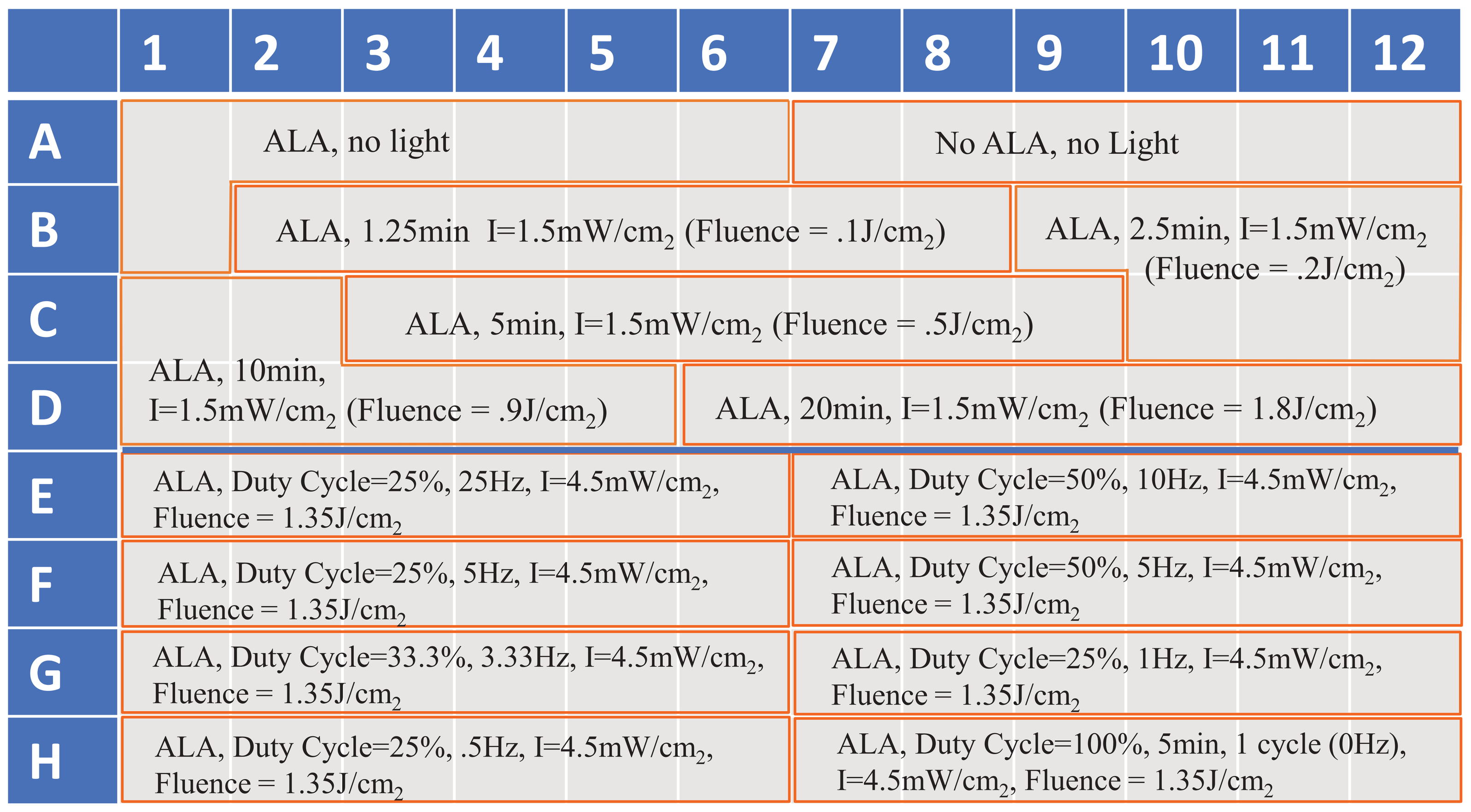

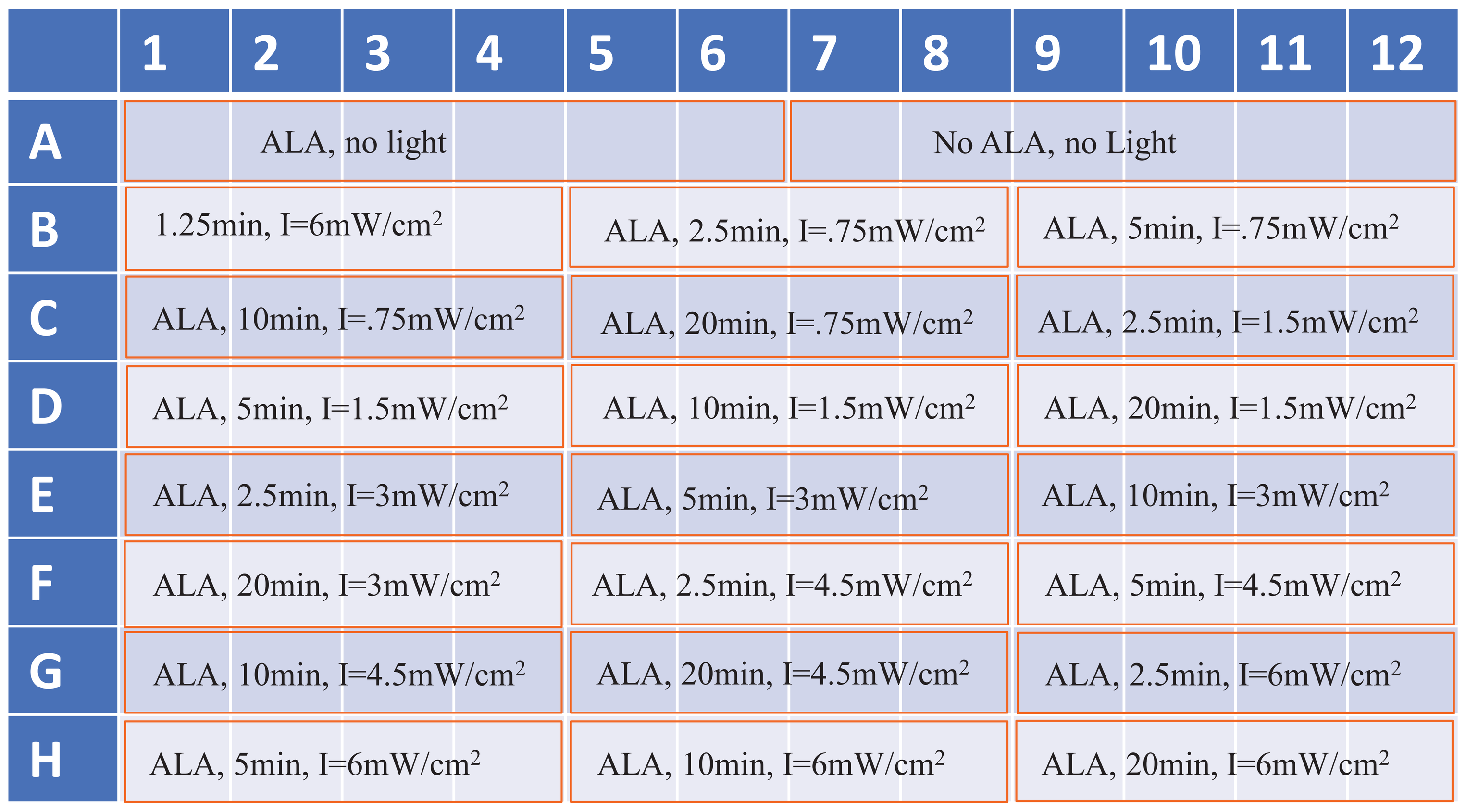

Duration	0	150	300	150	75	600	300	150	150	1200	600	300	150	300	1200	600	300	600	1200	600	1200	1200	Seconds
Irradiance	0	0.75	0.75	1.5	6	0.75	1.5	3	4.5	0.75	1.5	3	6	4.5	1.5	3	6	4.5	3	6	4.5	6	mW/cm^2
Fluence	0	0.1125	0.225	0.225	0.45	0.45	0.45	0.45	0.675	0.9	0.9	0.9	0.9	1.35	1.8	1.8	1.8	2.7	3.6	3.6	5.4	7.2	J/cm^2

Figure 5G. Below are the two microplate configurations used in the Pulsation graph: the bottom half of the "Increasing Duration"and "Increasing Irradiance" configurations. Only the bottom half of the 96-well plate (rows E-H) were used, containing parameters which keep the fluence equal at 1.35J/cm2 and the Irradiance equal t 4.5mW/cm2 while varying the proportion of time in the On and Off state as well as the number of cycles (thus modulating the duty cycle and frequency). For this graph, 96-well Experiments used in the comparison are: batch/plate id's: **2487**, 2438, 2439, 2477. The "Increasing Duration/Pulsation" batch configuration used is ighlighted in bold while the 3 "Increasing Irradiance/Pulsation" experiments are not.

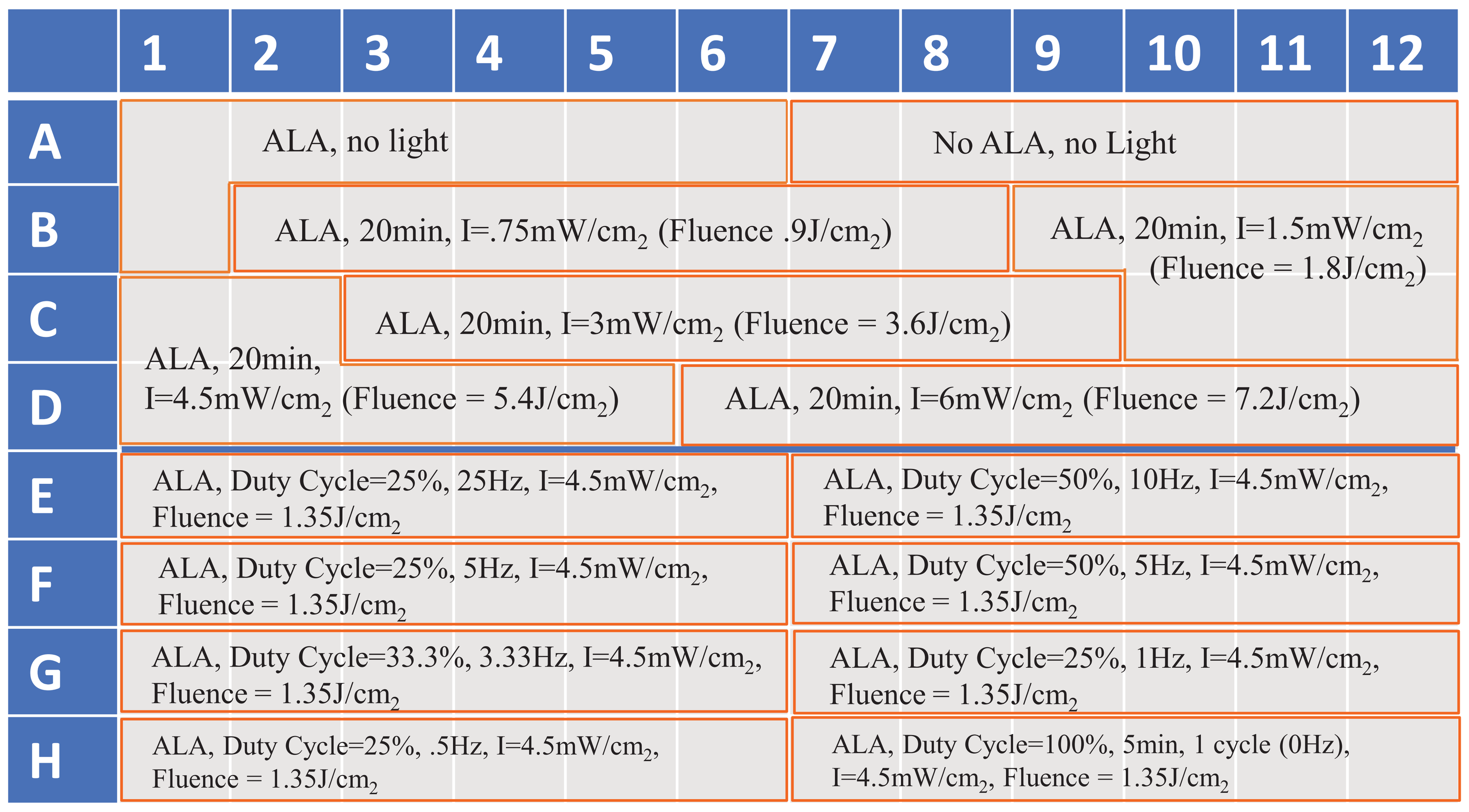

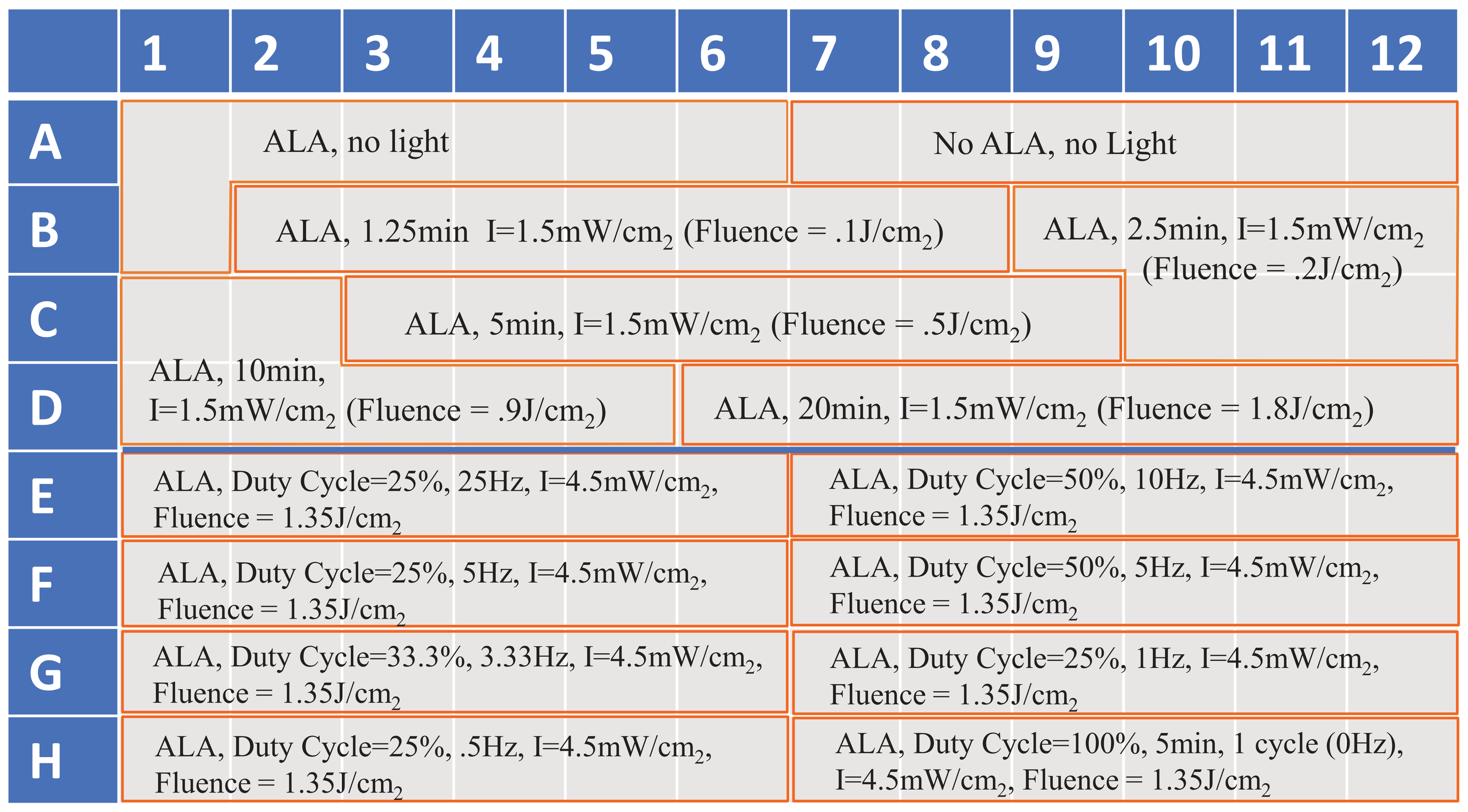

S4. Microplate Configurations/batches used in Figure 6 graphs.

Below are the two microplate configurations used in the **Figure 6A, 6B, and 6C** graphs which are used to show PpIX photo-bleaching with increasing fluence (by increasing duration at a fixed irradiance of 1.5mW/cm2). 96-well Experiments used in the comparison are:

6h ALA incubation graph A: batch/plate id's: 2399, 2466, 2467;
24h ALA incubation graph B: batch/plate id's: 2368, 2435, 2476;
36h ALA incubation graph C: batch/plate id's: 2382, 2480, 2481.

	1	2	3	4	5	6	7	8	9	10	11	12
A	ALA, no light						No ALA, no Light					
B	1.25min, I=6mW/cm^2				ALA, 2.5min, I=.75mW/cm^2				ALA, 5min, I=.75mW/cm^2			
C	ALA, 10min, I=.75mW/cm^2				ALA, 20min, I=.75mW/cm^2				ALA, 2.5min, I=1.5mW/cm^2			
D	ALA, 5min, I=1.5mW/cm^2				ALA, 10min, I=1.5mW/cm^2				ALA, 20min, I=1.5mW/cm^2			
E	ALA, 2.5min, I=3mW/cm^2				ALA, 5min, I=3mW/cm^2				ALA, 10min, I=3mW/cm^2			
F	ALA, 20min, I=3mW/cm^2				ALA, 2.5min, I=4.5mW/cm^2				ALA, 5min, I=4.5mW/cm^2			
G	ALA, 10min, I=4.5mW/cm^2				ALA, 20min, I=4.5mW/cm^2				ALA, 2.5min, I=6mW/cm^2			
H	ALA, 5min, I=6mW/cm^2				ALA, 10min, I=6mW/cm^2				ALA, 20min, I=6mW/cm^2			

Duration	0	150	300	150	75	600	300	150	150	1200	600	300	150	300	1200	600	300	600	1200	600	1200	1200	Seconds
Irradiance	0	0.75	0.75	1.5	6	0.75	1.5	3	4.5	0.75	1.5	3	6	4.5	1.5	3	6	4.5	3	6	4.5	6	mW/cm^2
Fluence	0	0.1125	0.225	0.225	0.45	0.45	0.45	0.45	0.675	0.9	0.9	0.9	0.9	1.35	1.8	1.8	1.8	2.7	3.6	3.6	5.4	7.2	J/cm^2
